# Exploring ASD Identification Using Different Types of Eye-Tracking Features

**DOI:** 10.3390/jemr19050111

**Published:** 2026-10-02

**Authors:** Kun Zhang, Shuo Zhang, Junling Kong, Jingying Chen

**Affiliations:** 1 National Engineering Research Center for E-Learning, Faculty of Artificial Intelligence in Education, Central China Normal University, Wuhan 430079, China; zhk@mail.ccnu.edu.cn (K.Z.); zhangshuo111@mails.ccnu.edu.cn (S.Z.);; 2 National Engineering Research Center for Educational Big Data, Faculty of Artificial Intelligence in Education, Central China Normal University, Wuhan 430079, China

**Keywords:** autism spectrum disorder, eye tracking, machine learning, dynamic and static multiscale feature fusion, participant-independent data partitioning

## Abstract

Eye tracking offers a non-invasive way of examining social visual attention in children and may support the objective identification of autism spectrum disorder (ASD). However, small-sample studies are often affected by individual differences, inconsistent feature selection, and limited integration of different gaze representations. This study explored a dynamic and static multiscale feature fusion framework for classifying children with pre-existing clinical ASD diagnoses and typically developing (TD) children using eye-tracking data collected during an emotion recognition task. After quality screening, 44 children aged 4–7 years were included (18 ASD and 26 TD). Static statistical features, dynamic sequences, early feature concatenation, and multiscale fusion were evaluated using participant-independent data partitioning, with static feature selection restricted to the training participants in each split. CNN–LSTM with multiscale fusion achieved an accuracy of 0.78 ± 0.08, ASD recall of 0.65 ± 0.22, F1-score of 0.71 ± 0.12, and AUC of 0.76 ± 0.16. A fully nested leave-one-participant-out evaluation provided additional internal validation of participant-level classification. Overall, the results indicate that different approaches to combining static and dynamic gaze representations may lead to different classification outcomes, while further validation in larger independent samples is needed.

## 1. Introduction

Autism spectrum disorder is a neurodevelopmental condition that emerges in early childhood and is primarily characterized by persistent difficulties in social communication and interaction, together with restricted and repetitive patterns of behavior, interests, or activities [1,2,3]. The reported prevalence of ASD among children has increased steadily in recent years. According to the US Centers for Disease Control and Prevention surveillance data, the estimated prevalence among 8-year-old children rose from 1 in 44 in 2018 to 1 in 31 in 2022 [4]. This upward trend has reinforced the importance of early identification and intervention, as timely intervention may improve outcomes in social communication, adaptive functioning, and cognitive development [5,6]. Despite this growing need, ASD assessment in clinical practice still relies largely on behavioral observation, standardized diagnostic instruments, developmental history, and the overall judgment of trained professionals [7,8]. Consequently, the assessment process may be influenced by examiner experience and clinical expertise. In addition, the marked heterogeneity of ASD, including variation in symptom profiles, severity, and developmental trajectories, poses further challenges to objective identification and limits the generalizability of data-driven models [9].

Eye tracking provides a non-invasive approach to investigating visual attention allocation, social cue processing, and information selection. As gaze behavior can be recorded continuously and quantified with relatively high precision, it has attracted increasing interest as a potential objective measure in ASD research [10]. Previous studies have identified differences between individuals with ASD and typically developing individuals in face viewing, attention to the eye region, joint attention, and patterns of visual exploration [11,12]. Taken together, these findings indicate that gaze behavior may capture meaningful differences in the processing of social information associated with ASD.

With the increasing use of machine learning, eye-tracking recordings have been transformed into a range of quantifiable features for ASD-related analysis. When viewing social videos or facial stimuli, children with ASD often differ from typically developing children in the amount of time spent fixating on socially relevant regions, such as the eyes, mouth, face, and body. These findings indicate that fixation measures derived from predefined areas of interest can capture differences in social attention associated with ASD [13]. Because such measures have relatively clear behavioral interpretations, they can also help explain how children with ASD distribute attention across social regions and explore visual scenes [14]. Beyond manually derived statistical measures, gaze sequences are often represented as spatial features, including scanpath images, heatmaps, and fixation maps. These representations allow deep learning models to learn patterns of fixation distribution and visual exploration directly from the data [15]. By converting complex gaze behavior into intuitive two-dimensional forms, they have been applied in studies of ASD screening and classification [16]. Compared with conventional statistical features, spatial representations preserve more information about the overall distribution of gaze across a visual scene. However, they remain less effective at characterizing continuous temporal variation and short-term transitions within the gaze sequence [17]. Thus, both static statistical measures and two-dimensional spatial representations compress the temporal structure of eye movements to some extent, which may limit their ability to capture how gaze behavior unfolds over time.

Previous sequence-modeling studies have represented consecutive fixations and saccades as text-like sequences and applied natural language processing methods to identify sequential patterns in eye movements. This approach has provided a useful basis for ASD identification from gaze-event sequences [18]. Behavioral research has also revealed differences in the temporal dynamics of social attention between children with ASD and TD children, suggesting that measures based only on total fixation duration may overlook shifts in attention allocation across different viewing phases [19]. Further studies have shown that continuous gaze sequences contain dynamic behavioral information relevant to ASD classification [20]. Dynamic gaze sequences can therefore describe how visual attention develops and changes over time. However, most existing sequence-based methods focus primarily on the order of fixation events and their short-range transitions. As a result, they may not fully capture the broader organization of visual attention. Their performance may also be affected by irregular intervals and varying durations between gaze events, difficulties in modeling long-range dependencies, and a high risk of overfitting when only small datasets are available.

Recently, eye-tracking research has increasingly integrated multi-source features. For example, CNN-LSTM models have been used to process eye-tracking data. CNNs extract spatial features from fixation distributions or visual stimuli, whereas LSTMs capture temporal dependencies in continuous fixation sequences. The resulting features are then fused through a fully connected layer for ASD identification [21]. In scanpath modeling, temporal information, pupil velocity, and spatial features are jointly encoded, and recurrent neural networks are used to model gaze dynamics. This approach captures both the spatial distribution and temporal dynamics of visual attention [22].

Other studies have jointly constructed fixation maps and distance sequences between consecutive fixation points and then fed them into classification models through feature concatenation. This approach achieves shallow fusion of multi-source gaze information [23]. Some methods encode gaze sequences and spatial saliency maps separately and subsequently fuse them at the feature level. Such fusion exploits the complementarity of different features to improve ASD identification performance [24].

Although existing studies have achieved multi-source gaze information fusion to some extent, most focus on the joint modeling of temporal and spatial features. The complementary relationship between dynamic sequence behaviors and static global features remains insufficiently explored. Fusion is often limited to simple concatenation, while gaze information at different levels is processed separately. Consequently, cross-feature interactions are not explicitly modeled, and a multilevel deep fusion framework has not yet been established.

Meanwhile, existing eye-tracking and machine learning studies on ASD identification vary considerably in sample size, task paradigm, feature construction, and validation strategy. Insufficient external validation limits the stability and generalizability of the findings [25,26]. Individuals with ASD also show substantial heterogeneity in symptom presentation and developmental trajectories, which further increases the difficulty of cross-subject identification [9]. Therefore, it is necessary to explore deep interactive fusion mechanisms between static features and dynamic sequences under a unified modeling framework to support more integrated and interpretable ASD-related classification in small-sample settings.

In this study, we collected eye-tracking data from children with ASD and TD children aged 4–7 years during an emotion recognition task. We aimed to explore a dynamic and static multiscale feature fusion framework for classifying children with pre-existing clinical ASD diagnoses and TD children using small-sample eye-tracking data while maintaining both classification performance and interpretability. This study systematically compared static statistical features, dynamic sequences, early feature concatenation, and dynamic and static multiscale feature fusion. It evaluated the applicability of these feature representations to participant classification and examined whether the proposed multiscale fusion framework could achieve more balanced performance than early feature concatenation.

## 2. Materials and Methods

### 2.1. Participants

This study recruited 55 children aged 4–7 years from Wuhan and Xianning, China, including 25 children with ASD and 30 TD children. Children in the ASD group had received clinical diagnoses from qualified medical professionals at hospitals or specialized autism institutions prior to recruitment, using standardized assessment tools including the ADI-R and ADOS-G. After screening, 11 children (7 ASD and 4 TD) were excluded because they did not complete the experimental procedure or because of substantial eye-tracking data loss. Consequently, 44 participants were included in the final analysis, comprising 18 children with ASD and 26 TD children. As shown in Table 1, the two groups did not differ significantly in age or gender distribution, indicating that they were comparable on these demographic characteristics.

### 2.2. Task and Eye-Tracking Procedure

**Figure 1:**
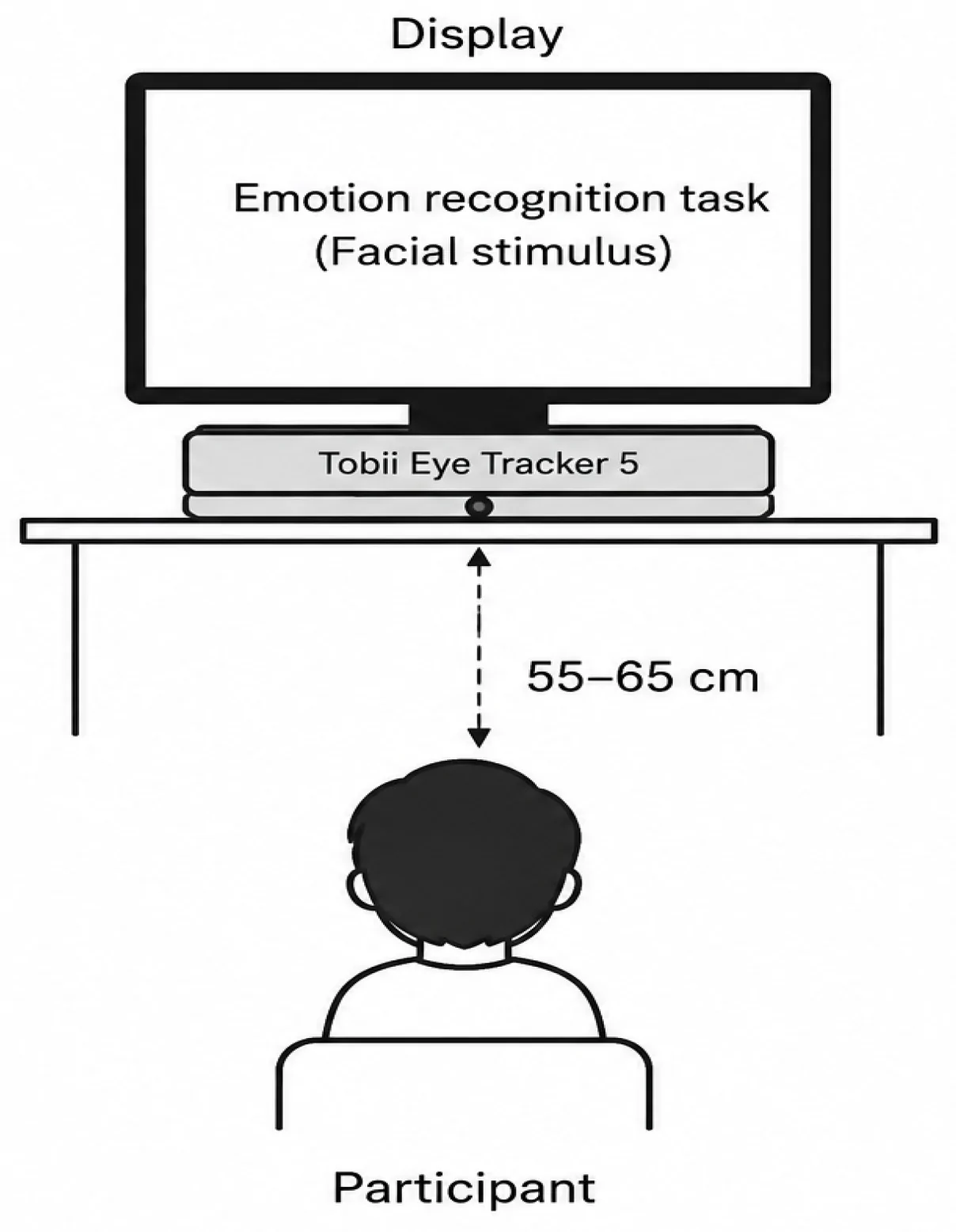
Schematic illustration of the experimental setup.

Experimental stimuli are designed based on common paradigms in emotion face recognition studies. The stimuli are also adapted to meet the needs of this study. Face images come from public image databases. These images are then uniformly cropped, resized, and adjusted for background and emotional intensity. This ensures good visual consistency and comparability across different stimuli. Stimuli include adult faces and child faces. Each type includes four basic emotions. These emotions are happy, sad, angry, and fear. Each emotion has two intensity levels. The levels are strong and weak. In total, there are 32 experimental trials. The facial stimulus remained on the screen until the completion of the current trial. A 1 s blank screen was presented between consecutive trials. Each child observed the facial expression and selected the corresponding emotion category from the four options. If the initial response was incorrect, the system provided graded cues across four predefined levels. The first incorrect response triggered Level 1, in which key facial regions were visually highlighted. If the child responded incorrectly again, Level 2 was provided, combining the visual highlighting of key facial regions with an audio cue. A third incorrect response triggered Level 3, in which the child was explicitly informed of the emotion category. If the response remained incorrect, Level 4 was provided, in which the correct response option flashed in red. The child made another response after each cue, and the cueing procedure continued until the correct response was made or all four cue levels had been presented. The experiment was conducted in a quiet, child-familiar setting in kindergartens or special education institutions. The experimental setup is illustrated in Figure 1.

The program ran on a computer with a resolution of 2560 × 1600 pixels. Eye-tracking data were recorded using a Tobii Eye Tracker 5 (Tobii AB, Danderyd, Sweden). The sampling rate was 33 Hz. Participants were seated approximately 55–65 cm from the display. Before the experimental task, each participant underwent an individual seven-point calibration using Tobii Experience. The seven calibration targets consisted of one target at the center of the screen, three targets in the upper region (left, center, and right), and three targets in the lower region (left, center, and right). Each calibration target required 3 s of continuous fixation before proceeding to the next target. After all seven calibration targets had been successfully fixated, the system automatically indicated that the calibration was successful. If the calibration was unsuccessful, the seven-point calibration procedure was repeated. The formal experimental task began only after successful completion of the calibration procedure.

During the experiment, two experimenters were responsible for procedure explanation, equipment operation, and data recording. Informed consent was obtained from the legal guardians of all children. The study strictly followed ethical guidelines, with all personal information and experimental data kept confidential.

### 2.3. Data Preprocessing

Trials with an effective gaze-sampling proportion below 65% were excluded from subsequent analysis. After data-quality screening, 620 valid trials were retained. The proportions of excluded trials were 58.5% in the ASD group and 54.2% in the TD group, with no significant difference between groups (χ^2^(1) = 2.38, *p* = 0.123). At the participant level, the mean number of retained valid trials was 13.28 ± 7.35 in the ASD group and 14.65 ± 5.15 in the TD group. These trials were used for participant-level probability aggregation, with the number of contributing trials varying across participants. The continuous gaze recordings from the retained trials were segmented according to the timestamps of the graded cues, with each cue stage treated as a separate segment for subsequent feature extraction and sequence modeling, yielding 977 gaze segments.

Raw gaze data were first checked for coordinate validity, with coordinates outside the screen boundaries treated as invalid. Short gaps of up to eight consecutive samples were linearly interpolated, followed by Savitzky–Golay smoothing using a seven-sample window and a second-order polynomial. Samples remaining invalid after interpolation were treated as tracking loss. Blinks were not identified separately, and unavailable samples during blinks or other temporary tracking failures were treated as missing data. The preprocessed gaze coordinates were normalized by screen width and height and mapped to predefined regions of interest (ROIs), including the face, eyes, mouth, option area, and other regions. Fixation candidates were identified by grouping consecutive samples with normalized inter-sample displacement below 0.03, and only segments lasting at least 100 ms were retained as fixation events. Saccades were not independently identified using a separate velocity-threshold detector. Static feature extraction and selection are described in Section 2.4.1. During sequence preprocessing, gaze segments of approximately 3–7 s were retained. A maximum sequence length of 211 sampling points was adopted as a practical standardization setting rather than an empirically optimized value, corresponding to approximately 6.4 s at 33 Hz and thus lying near the upper end of the retained segment-duration range. Longer segments were truncated to the first 211 points.

At the model-input stage, residual missing values in the static feature matrix were replaced with 0; for dynamic sequences, missing values were first forward-filled and any remaining values were set to 0. Shorter sequences were zero-padded only after the last observed sampling point to reach 211 points. An additional sequence-length sensitivity analysis using a maximum length of 165 sampling points was conducted under the exploratory feature specification, with all other modeling settings unchanged.

### 2.4. Gaze Features

#### 2.4.1. Static Statistical Features

Static statistical features were extracted from the preprocessed gaze data to characterize overall gaze distribution, fixation behavior, gaze movement, and transitions between predefined ROIs. Candidate features were derived from gaze coordinates, detected fixation events, and ROI-based viewing patterns. Given the sampling rate of 33 Hz, gaze-movement measures were treated as coarse descriptors of visual exploration rather than precise measures of rapid oculomotor dynamics. Fixation-to-fixation transition measures were computed from successive detected fixations rather than independently detected saccade events. For each participant-level split, static feature selection was performed using only the training participants. Candidate features were first screened for data completeness and group differences using Hedges’ g. Spearman correlation analysis was then used to reduce redundancy among highly correlated features, with up to seven features retained for subsequent modeling. Because feature selection was conducted independently for each split, the retained static feature set could vary across evaluations. The features selected in each of the five repeated evaluations are provided in Supplementary Table S1. A fixed seven-feature specification derived from the full dataset was retained only for the exploratory analyses, with the specific features and their corresponding definitions provided in Supplementary Table S2.

#### 2.4.2. Dynamic Sequences

Dynamic sequences were constructed to preserve the continuous temporal characteristics of gaze behavior. Each sampling point was represented by eight features: normalized horizontal and vertical gaze coordinates, gaze movement between consecutive sampling points, and five binary ROI indicators corresponding to the face, eyes, mouth, selection area, and other regions. The horizontal and vertical coordinates were normalized by screen width and height, respectively. Gaze movement was calculated as the Euclidean distance between consecutive gaze positions in the normalized coordinate space:

$$m_{t}=\sqrt{{\left(x_{t}^{\mathrm{norm}}-x_{t-1}^{\mathrm{norm}}\right)}^{2}+{\left(y_{t}^{\mathrm{norm}}-y_{t-1}^{\mathrm{norm}}\right)}^{2}}$$

where $x_{t}^{\mathrm{norm}}$ and $y_{t}^{\mathrm{norm}}$ denote the normalized horizontal and vertical gaze coordinates at sampling point t, respectively. The gaze movement value of the first sampling point in each sequence was set to 0.

The ROI boundaries were determined according to the displayed facial stimulus and response-option locations on the screen. The face indicator was set to 1 when the gaze position fell within the face region, including the eye and mouth subregions, while the eye and mouth indicators separately identified these subregions. The selection indicator was set to 1 for gaze positions within the response-option area, and valid gaze positions outside these predefined ROIs were assigned to the other category.

The resulting eight-dimensional feature vectors were arranged chronologically to construct each dynamic gaze sequence and organized into a three-dimensional tensor:

$$X_{\mathrm{seq}}\in R^{N\times T\times 8}$$

where T denotes the sequence length.

#### 2.4.3. Early Feature Concatenation

Early feature concatenation is constructed as a comparison to validate the proposed fusion framework. This method uses sequence models to extract features from dynamic sequences. This produces dynamic features ${\;F}_{d}$. Meanwhile, static statistical measures are transformed through feature mapping. This produces static features $F_{s}$. These two features are directly concatenated along the feature dimension. This forms a unified fused feature vector. The vector is then fed into a classifier for ASD identification:

(1) $${\;\;\;\;\;\;F}_{d}=f_{\mathrm{dyn}}(X_{\mathrm{seq}})$$

(2) $${\;F}_{s}=f_{\mathrm{stat}}(X_{\mathrm{tab}})\;$$

(3) $${\;F}_{\mathrm{concat}}=[F_{d};F_{s}]$$

(4) $$\hat{y}=\mathrm{Softmax}(W_{c}F_{\mathrm{concat}}+b_{c})\;$$

$W_{c}$ represents the weight matrix of the classifier, and $b_{c}$ represents the bias vector. $\hat{y}$ represents the class predicted by the model.

#### 2.4.4. Dynamic and Static Multiscale Feature Fusion Framework

**Figure 2:**
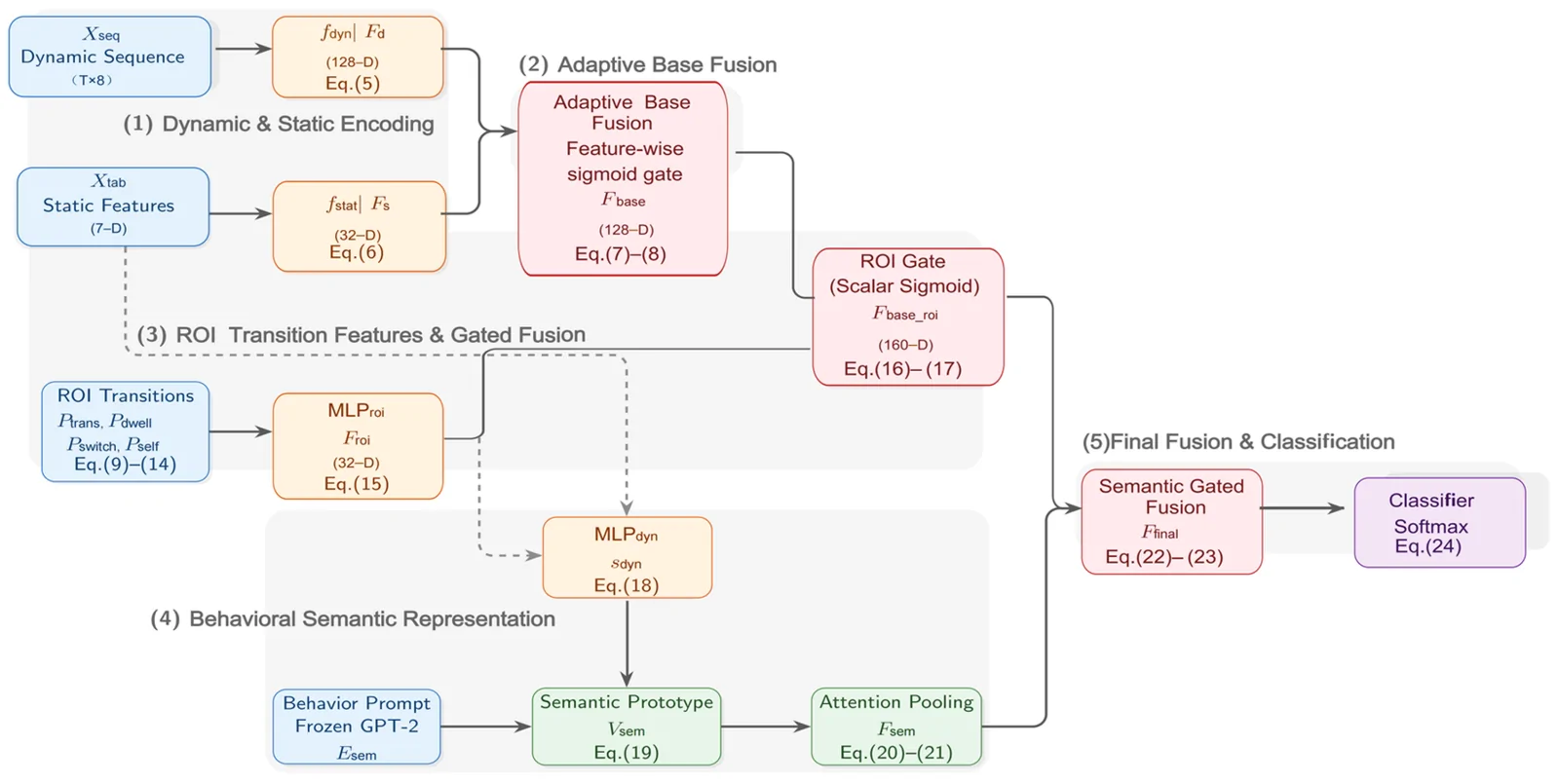
Architecture of the proposed dynamic and static multiscale feature fusion model. Solid arrows indicate feature flow; dashed arrows indicate modulation.

To achieve multi-level fusion of different eye-tracking features, this paper proposes a dynamic and static multiscale feature fusion framework, as Figure 2. The multiscale fusion models share the same overall fusion framework, with the main difference lying in the dynamic sequence encoder. The framework first encodes dynamic sequences and static features separately to preserve temporal behavioral information and global visual attention patterns. On this basis, an ROI transition structure is further constructed from dynamic sequences. This structure captures the movement patterns of visual attention across different ROIs. Behavioral semantic representations are also introduced. This enhances high-level behavioral abstraction capability. Finally, dynamic-static fusion features, ROI transition features, and behavioral semantic features are jointly modeled to form a complete multi-layer fused representation. Unlike early feature concatenation, which directly stacks heterogeneous features and may overlook differences in feature importance or introduce redundant information, the proposed framework achieves information fusion through hierarchical encoding and structured modeling. This allows information at different levels to complement and enhance each other within a unified framework and facilitates the capture of key behavioral patterns.

(1) Dynamic and Static feature encoding

This study first encodes dynamic sequences and static statistical features separately to preserve information from different types of behavioral features. Across the evaluated sequence encoders, the dynamic branch produced a 128-dimensional representation. The seven-dimensional static feature vector was encoded into a 32-dimensional representation using a fully connected layer with ReLU activation and dropout of 0.2.

(5) $${\;\;F}_{d}=f_{\mathrm{dyn}}(X_{\mathrm{seq}})\;$$

(6) $$F_{s}=f_{\mathrm{stat}}(X_{\mathrm{tab}})\;$$

$X_{\mathrm{seq}}$ represents dynamic sequences. $X_{\mathrm{tab}}$ represents static statistical features. $f_{\mathrm{dyn}}(\cdot )$ and $f_{\mathrm{stat}}(\cdot )$ are dynamic and static feature encoders, respectively.

(2) Adaptive Base Fusion mechanism

To avoid information redundancy caused by early feature concatenation, an adaptive fusion mechanism is introduced.

(7) $$\alpha_{\mathrm{base}}=\sigma (W_{\mathrm{base}}[F_{d};F_{s}]+b_{\mathrm{base}})\;$$

(8) $${\;\;F}_{\mathrm{base}}=\alpha_{\mathrm{base}}⊙F_{d}+(1-\alpha_{\mathrm{base}})⊙F_{s}\;$$

[·;·] denotes feature concatenation. σ represents Sigmoid. ⊙ denotes element-wise multiplication.

The dynamic and static representations were separately projected into a common 128-dimensional space using fully connected layers with ReLU activation and dropout of 0.3. The projected features were concatenated and passed through a gating network with a 64-unit ReLU hidden layer to generate a 128-dimensional feature-wise sigmoid gate, which adaptively weighted the two representations to produce the 128-dimensional base fusion representation.

(3) ROI transition features and gated fusion

To compensate for the limitations of dynamic sequences in expressing spatial structure, the model explicitly extracts spatial transition patterns and temporal retention features between ROIs. Let $R_{t}\in \{0,1{\}}^{N}$ be a binary ROI status vector at time step t. Here, *N* is the total number of ROIs (face, eyes, mouth, etc.). Let $T_{v}$ be the total number of time steps with valid ROI detection. The feature extraction process is as follows:

ROI transitions were determined between consecutive samples with valid ROI assignments. Samples within the same ROI were treated as self-transitions, whereas changes between ROIs were counted as switches. Residual missing ROI indicators were first forward-filled and then set to 0 if still missing; time points with no active ROI indicator were treated as invalid states and excluded from transition counting.

A. Region transition probability matrix $P_{\mathrm{trans}}$

First, the raw number of gaze transitions from ROIi to ROIj is counted as $C_{ij}$:

(9) $$C_{ij}=\sum_{t=1}^{T-1}I(R_{t-1}=i\wedge R_{t}=j)$$

This is then globally normalized to obtain the standardized region transition probability matrix:

(10) $$P_{\mathrm{trans}}=\frac{C_{ij}}{\sum_{m=1}^{N}\sum_{n=1}^{N}C_{mn}}$$

B. ROI dwell proportion $P_{\mathrm{dwell}}$

The proportion of gaze dwell time in each ROI is calculated against the total valid time:

(11) $$P_{\mathrm{dwell},i}=\frac{\sum_{t=1}^{T}I(R_{t}=i)}{T_{v}}$$

C. Region switch rate $P_{\mathrm{switch}}$

This measures how often gaze shifts between different ROIs:

(12) $${\;\;\;P}_{\mathrm{switch}}=\frac{\sum_{t=1}^{T-1}I(R_{t-1}\neq R_{t})}{T_{v}-1}$$

D. Self-transition proportion $P_{\mathrm{self}}$

This measures the proportion of time that gaze remains in the current ROI without switching:

(13) $$P_{\mathrm{self}}=\frac{\sum_{i=1}^{N}C_{ii}}{\sum_{m=1}^{N}\sum_{n=1}^{N}C_{mn}}$$

E. Spatial structure feature generation and gated mechanism

The above statistical features are flattened and concatenated into an explicit transition vector $v_{\mathrm{roi}}$, which is then passed through a nonlinear mapping to obtain the spatial feature $F_{\mathrm{roi}}$:

(14) $$v_{\mathrm{roi}}=[\mathrm{Flatten}(P_{\mathrm{trans}});P_{\mathrm{dwell}};P_{\mathrm{switch}};P_{\mathrm{self}}]\;$$

(15) $$F_{\mathrm{roi}}={\mathrm{MLP}}_{\mathrm{roi}}(v_{\mathrm{roi}})\;$$

The transition vector was encoded into a 32-dimensional ROI transition representation using a multilayer perceptron with a 64-unit hidden layer, ReLU activation, and dropout of 0.3. A transition feature gate is then introduced to adaptively control the degree to which the spatial structure features supplement the basic behavioral features.

(16) $$g_{\mathrm{roi}}=\sigma (W_{\mathrm{roi}}[F_{\mathrm{base}};F_{\mathrm{roi}}]+b_{\mathrm{roi}})\;$$

(17) $$F_{\mathrm{base}\_\mathrm{roi}}=[F_{\mathrm{base}};g_{\mathrm{roi}}\cdot F_{\mathrm{roi}}]\;$$

The ROI transition gate used a 64-unit ReLU hidden layer with dropout of 0.3 and a scalar sigmoid output to control the contribution of the 32-dimensional ROI transition representation, which was then concatenated with the 128-dimensional base fusion representation to form a 160-dimensional representation.

(4) Behavioral semantic representation

To introduce high-level behavioral representations into the fusion framework, behavioral concept prompts were used to initialize fixed semantic prototypes. The language model embeddings served as semantic prototypes rather than an independent data modality. Recent studies have demonstrated the potential of pretrained language model semantic spaces for linking numerical time-series representations with higher-level semantic information [27]. Here, the prompts described interpretable gaze patterns corresponding to the eye-tracking features, providing a semantic reference space for these low-level behavioral characteristics. To incorporate individual-specific information, the fixed semantic prototypes were dynamically modulated using static statistical and ROI transition features, thereby mapping low-level features into a unified semantic space:

(18) $$s_{\mathrm{dyn}}={\mathrm{MLP}}_{\mathrm{dyn}}([X_{\mathrm{tab}};F_{\mathrm{roi}}])$$

(19) $$V_{\mathrm{sem}}=\phi (E_{\mathrm{sem}}+\lambda \cdot s_{\mathrm{dyn}})\;$$

$E_{\mathrm{sem}}=f_{\mathrm{GPT}}\left(\mathrm{prompt}\right)$ is the static semantic prototype matrix obtained by encoding fixed behavioral prompts using a frozen pre-trained large language model (GPT-2). $s_{\mathrm{dyn}}$ is the dynamic behavioral state derived from the current individual features. λ is the scaling factor for state modulation, which was fixed at 0.5 in this study.

Specifically, eight fixed behavioral concept prompts were encoded using the pretrained GPT-2 base model (“gpt2”; embedding dimension = 768): “reduced eye fixation during social visual attention”, “increased mouth fixation during face observation”, “frequent gaze transitions between eyes and mouth”, “high attention to non-social or other regions”, “unstable scan path velocity during viewing”, “delayed first fixation on face regions”, “high gaze switching frequency across regions of interest”, and “stable social attention with sustained face and eye fixation”. GPT-2 was used only to generate fixed semantic prototype embeddings and was not fine-tuned during training.

The dynamically modulated 768-dimensional embeddings were projected into a 128-dimensional semantic space using a fully connected layer with GELU activation, dropout of 0.3, and layer normalization. Attention pooling was then applied to the semantic tokens $V_{\mathrm{sem}}$ to obtain a compact 128-dimensional behavioral semantic feature $F_{\mathrm{sem}}$:

(20) $$\alpha_{\mathrm{sem}}=\mathrm{Softmax}(W_{\mathrm{attn}}V_{\mathrm{sem}})\;$$

(21) $$F_{\mathrm{sem}}=\sum (\alpha_{\mathrm{sem}}⊙V_{\mathrm{sem}})\;$$

(5) Final fusion and classification

After obtaining the composite spatial structural features ${\;F}_{\mathrm{base}\_\mathrm{roi}}$ and the high-level behavioral semantic features ${\;F}_{\mathrm{sem}}$, the model used a gated fusion mechanism to adaptively control the contribution of semantic features. The semantic gate consisted of a 64-unit ReLU hidden layer with dropout of 0.3 and a scalar sigmoid output, which weighted the 128-dimensional semantic representation before concatenation with the preceding 160-dimensional fused representation, producing a 288-dimensional global behavioral representation. The global behavioral feature $F_{\mathrm{final}}$ is then constructed through dynamic concatenation:

(22) $$g_{\mathrm{sem}}=\sigma (W_{\mathrm{sem}}[F_{\mathrm{base}\_\mathrm{roi}};F_{\mathrm{sem}}]+b_{\mathrm{sem}})\;$$

(23) $$F_{\mathrm{final}}=[F_{\mathrm{base}\_\mathrm{roi}};g_{\mathrm{sem}}\cdot F_{\mathrm{sem}}]$$

After obtaining the final global representation $F_{\mathrm{final}}$, it is fed into a classifier to identify ASD and TD:

(24) $$\hat{y}=\mathrm{Softmax}(W_{c}F_{\mathrm{final}}+b_{c})\;$$

The classifier used a 64-unit ReLU hidden layer with dropout of 0.3, followed by a two-unit output layer for ASD/TD classification.

### 2.5. Models and Evaluation

To compare the classification performance of different eye-tracking feature representations, this study evaluated multiple classification models under different feature settings. Static statistical features were evaluated using RF, KNN, SVM, LR, and XGBoost. Dynamic sequences, early feature concatenation, and dynamic and static multiscale feature fusion were evaluated using CNN, BiLSTM, TCN, and CNN-LSTM. The deep learning models were trained using cross-entropy loss and the Adam optimizer with a learning rate of 1 × 10^−3^, weight decay of 1 × 10^−4^, and a batch size of 32 for up to 80 epochs. Dropout was used for regularization, with early stopping based on validation AUC and a patience of 12 epochs. The principal hyperparameters were predefined based on commonly used settings and modeling experience rather than exhaustive search. The pretrained GPT-2 parameters used for the behavioral semantic prototypes remained frozen during training.

The primary evaluation used repeated stratified participant-level random holdout validation. For each random seed, participants were divided into training, validation, and test sets at approximate ratios of 64%, 16%, and 20%, respectively. Across all five participant-level splits, the training, validation, and test sets contained 28 (11 ASD/17 TD), 7 (3 ASD/4 TD), and 9 (4 ASD/5 TD) participants, respectively. Stratification maintained the same group composition across splits, while all trials from each child remained within a single subset. The same participant partitions were used for all feature settings and model architectures. For feature settings involving static statistical features, feature selection was conducted within the training data of each participant-level split, without using validation or test data. Standardization parameters were estimated from the training data and applied unchanged to the validation and test sets. The validation set was used for model selection, early stopping, and classification-threshold determination, while the test set was used only for final evaluation.

Five random seeds (1–5) determined participant partitioning, model initialization, and training-data order. Thus, the reported mean and standard deviation reflect variability associated with both participant partitioning and stochastic model training. Each trained model generated an ASD probability for each valid trial. Probabilities from all valid trials of the same child were averaged to obtain a participant-level probability. The classification threshold determined from the validation set was then applied to the corresponding test set. Accuracy, ASD recall, F1-score, and AUC were calculated at the participant level.

As an additional internal validation, a fully nested leave-one-participant-out (LOPO) evaluation was conducted for the CNN-LSTM multiscale fusion model. In each outer fold, one of the 44 participants was held out for testing and was completely excluded from static feature selection, model training, and threshold determination. Static feature selection was performed using only the inner training participants, and neither the inner validation participants nor the outer held-out participant contributed to feature selection. Model training, early stopping, and threshold determination were completed using the corresponding inner training and validation data before evaluation on the outer held-out participant. Accuracy, ASD recall, F1-score, AUC, specificity, precision, NPV, the confusion matrix, and the Brier score were calculated from the 44 held-out participant-level predictions.

Exploratory analyses were also conducted using a fixed seven-feature specification established from the full dataset. These analyses included additional model comparisons, ablation analysis, and sequence-length sensitivity analysis.

## 3. Results

### 3.1. Static Statistical Feature Model Results

The results of static statistical feature models are shown in Table 2. RF achieved the highest mean accuracy of 0.76 ± 0.09, with an F1-score of 0.67 ± 0.19, indicating relatively balanced mean performance across the evaluated metrics. SVM achieved the highest AUC of 0.85 ± 0.20, with an accuracy of 0.73 ± 0.18 and an F1-score of 0.67 ± 0.24. SVM also showed the highest ASD recall, reaching 0.70 ± 0.33. LR and XGBoost showed relatively lower overall mean performance.

### 3.2. Dynamic Sequences Model Results

The results of the dynamic sequence models are shown in Table 3. CNN-LSTM achieved the highest mean accuracy of 0.78 ± 0.11 and the highest mean AUC of 0.84 ± 0.09. TCN achieved the highest ASD recall of 0.75 ± 0.18, with an accuracy of 0.71 ± 0.13 and an AUC of 0.82 ± 0.09. CNN achieved an accuracy of 0.76 ± 0.09 and an AUC of 0.82 ± 0.10, whereas BiLSTM showed lower mean accuracy and AUC values of 0.62 ± 0.10 and 0.55 ± 0.15, respectively.

### 3.3. Early Feature Concatenation Model Results

The results of the early feature concatenation models are presented in Table 4. For CNN, accuracy decreased from 0.76 ± 0.09 with dynamic sequences alone to 0.71 ± 0.15 after early feature concatenation, while ASD recall decreased from 0.70 ± 0.21 to 0.60 ± 0.29. For CNN-LSTM, accuracy decreased from 0.78 ± 0.11 to 0.76 ± 0.15, while ASD recall decreased from 0.65 ± 0.29 to 0.60 ± 0.29. BiLSTM showed higher mean accuracy, F1-score, and AUC after early feature concatenation, accompanied by a lower ASD recall, whereas TCN showed lower mean values across all four metrics. Overall, early feature concatenation did not show a consistent improvement over the corresponding dynamic sequence models.

### 3.4. Dynamic and Static Multiscale Feature Fusion Model Results

The results of the dynamic and static multiscale feature fusion models are presented in Table 5. CNN and CNN-LSTM achieved the highest mean accuracy of 0.78, with values of 0.78 ± 0.11 and 0.78 ± 0.08, respectively. CNN achieved the highest mean ASD recall of 0.70 ± 0.28. BiLSTM achieved the highest mean AUC of 0.81 ± 0.16, with an accuracy of 0.76 ± 0.18, ASD recall of 0.65 ± 0.22, and an F1-score of 0.71 ± 0.18. CNN-LSTM achieved an accuracy of 0.78 ± 0.08, ASD recall of 0.65 ± 0.22, an F1-score of 0.71 ± 0.12, and an AUC of 0.76 ± 0.16. Overall, performance varied across network architectures and evaluation metrics.

### 3.5. Further Evaluation of the Dynamic and Static Multiscale Feature Fusion

#### 3.5.1. Comparison with Early Feature Concatenation

To further evaluate the dynamic and static multiscale feature fusion framework, its performance was compared with early feature concatenation across the four network architectures. The mean performance differences between the two feature settings are presented in Table 6.

Multiscale fusion showed higher mean accuracy, F1-score, and AUC than early feature concatenation across all four network architectures. For CNN, the corresponding differences in accuracy, ASD recall, F1-score, and AUC were +0.07, +0.10, +0.08, and +0.02, respectively. For CNN-LSTM, the corresponding differences were +0.02, +0.05, +0.05, and +0.01. ASD recall remained unchanged for BiLSTM and TCN. Overall, the comparison showed a descriptive trend of higher mean performance under multiscale fusion, although the magnitude of the differences varied across network architectures and evaluation metrics.

Detailed paired performance differences and their corresponding 95% confidence intervals are provided in Supplementary Table S3. The 95% confidence intervals of the paired differences included zero, and paired comparisons across the five matched participant-level evaluations did not reach statistical significance (all *p* > 0.05). Therefore, the observed differences should be interpreted as descriptive performance trends rather than statistically confirmed improvements.

#### 3.5.2. Fully Nested Leave-One-Participant-Out Evaluation

A fully nested leave-one-participant-out (LOPO) evaluation was conducted for the CNN-LSTM multiscale fusion model as an additional participant-level evaluation. In each outer fold, the held-out participant was completely excluded from static feature selection, model training, and threshold determination. Static feature selection was performed using only the participants in the inner training set, and the selected features were subsequently used for model training before evaluation on the held-out participant. The participant-level performance is presented in Table 7.

Across the 44 participants, the fully nested LOPO evaluation achieved an accuracy of 0.75, ASD recall of 0.78, F1-score of 0.72, and AUC of 0.87. The corresponding specificity, precision, NPV, and Brier score were 0.73, 0.67, 0.83, and 0.163, respectively.

### 3.6. Comparison of the Four Features

To provide an overall comparison across the four eye-tracking feature settings, representative models were summarized for static statistical features, dynamic sequences, early feature concatenation, and dynamic and static multiscale feature fusion. RF was used to represent the static feature setting because it achieved the highest mean accuracy among the evaluated conventional machine-learning models. CNN-LSTM was used as the representative architecture for the three sequence-based feature settings, providing a consistent network architecture for comparison across dynamic sequences, early feature concatenation, and multiscale fusion. The results are summarized in Table 8.

Among the four representative settings, the dynamic-sequence CNN-LSTM and multiscale-fusion CNN-LSTM achieved the same highest mean accuracy of 0.78. The dynamic-sequence setting achieved the highest mean AUC of 0.84, while the multiscale-fusion setting achieved the highest mean F1-score of 0.71. ASD recall was 0.65 for the static, dynamic-sequence, and multiscale-fusion settings and 0.60 for early feature concatenation.

Within the CNN-LSTM architecture, multiscale fusion showed higher mean accuracy, ASD recall, F1-score, and AUC than early feature concatenation, with differences of +0.02, +0.05, +0.05, and +0.01, respectively. Compared with dynamic sequences alone, multiscale fusion showed the same mean accuracy and ASD recall, a higher mean F1-score (0.71 vs. 0.69), and a lower mean AUC (0.76 vs. 0.84).

Approximate descriptive 95% confidence intervals for the representative models across the four feature settings are provided in Supplementary Table S4. Several intervals, particularly those for ASD recall, were relatively wide across the five repeated participant-level evaluations.

### 3.7. Comparison with Previous Studies

To place the present results in the context of previous ASD eye-tracking research, Table 9 summarizes studies that used participant-independent evaluation. Additional information on participant characteristics, experimental tasks, eye-tracking systems, diagnostic procedures, and other performance metrics is provided in Supplementary Table S5. Because the studies differed in sample size, stimulus materials, feature representations, model architectures, and evaluation protocols, the reported performance values are presented for contextual comparison rather than direct ranking.

The mean classification accuracies obtained across the four representative feature settings in the present study ranged from 0.76 to 0.78. Specifically, RF with static statistical features achieved an accuracy of 0.76 ± 0.09, while CNN-LSTM achieved accuracies of 0.78 ± 0.11 with dynamic sequences, 0.76 ± 0.15 with early feature concatenation, and 0.78 ± 0.08 with dynamic and static multiscale feature fusion. These values were within the range of the accuracies reported in the previous participant-independent eye-tracking studies summarized in Table 9.

### 3.8. Interpretability Analysis of the Dynamic and Static Multiscale Feature Fusion

#### 3.8.1. Model-Derived Adaptive Gating Weight Analysis

**Figure 3:**
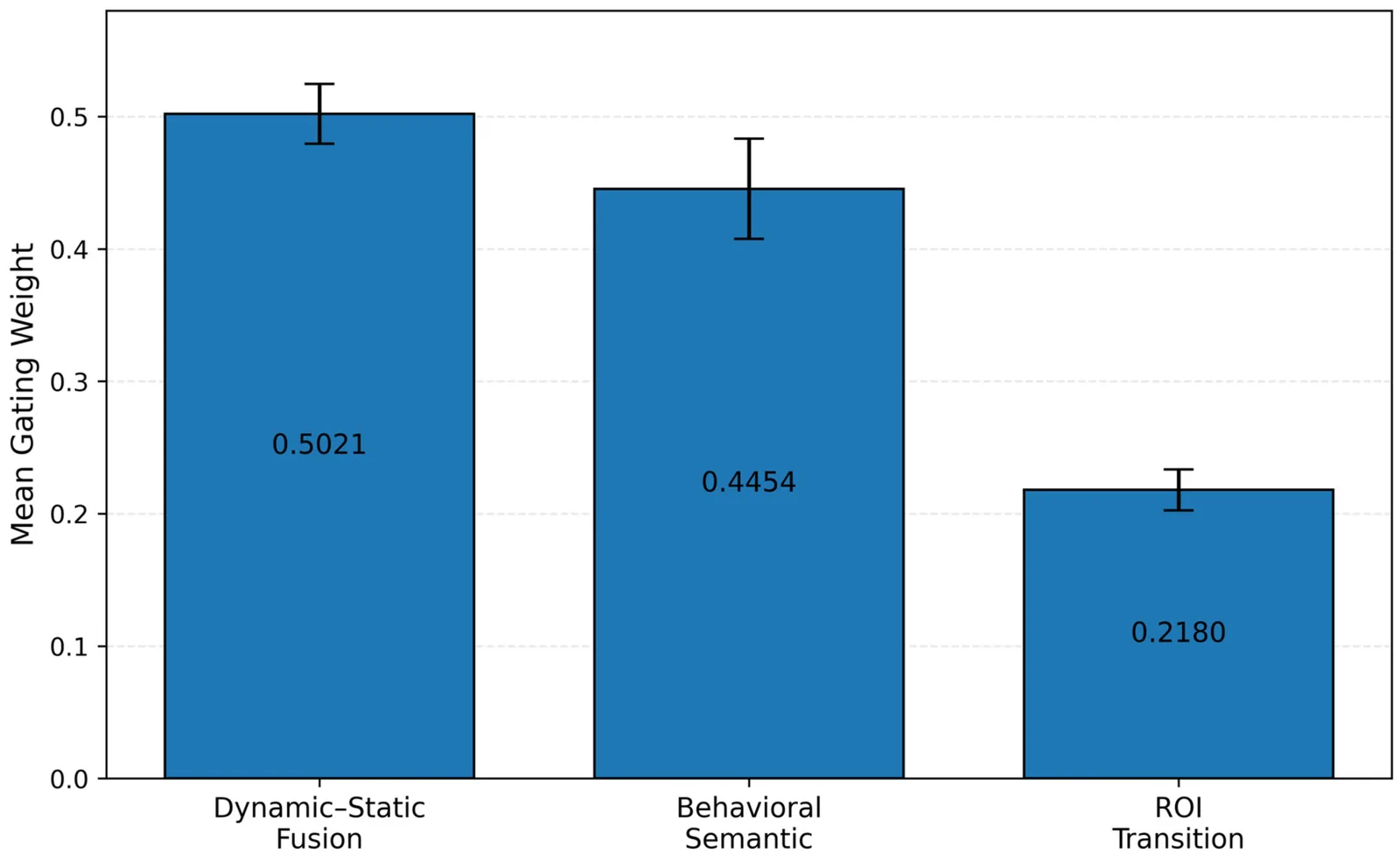
Model-derived adaptive gating weights across feature branches.

Based on the CNN-LSTM model with dynamic and static multiscale feature fusion, the mean gating weights of the different feature branches were further examined, as shown in Figure 3. For the dynamic–static fusion gate, the feature-wise sigmoid weights were first averaged across the 128 fusion dimensions for each test sample. For each participant-level split, the resulting gating weights were then averaged across ASD test samples. The split-level mean gating weights were subsequently summarized across the five random seeds and are reported as mean ± SD across the five participant-level splits. The mean gating weights were 0.5021 ± 0.0226 for the dynamic–static feature fusion branch, 0.4454 ± 0.0378 for the behavioral semantic branch, and 0.2180 ± 0.0155 for the ROI social visual attention transition branch. These results show that the model assigned different average weights to information from the three branches rather than combining them uniformly. The relatively higher mean weights of the dynamic–static fusion branch and the behavioral semantic branch indicate that the trained model assigned greater average weighting to these representations under the present experimental setting. The ROI social visual attention transition branch showed a lower but non-zero mean weight, suggesting that it was retained as supplementary information by the model. These gating weights are model-derived measures and should not be interpreted as direct evidence of causal contribution, explained variance, or feature importance.

#### 3.8.2. Model-Derived Temporal Attention Analysis of Dynamic Sequences

**Figure 4:**
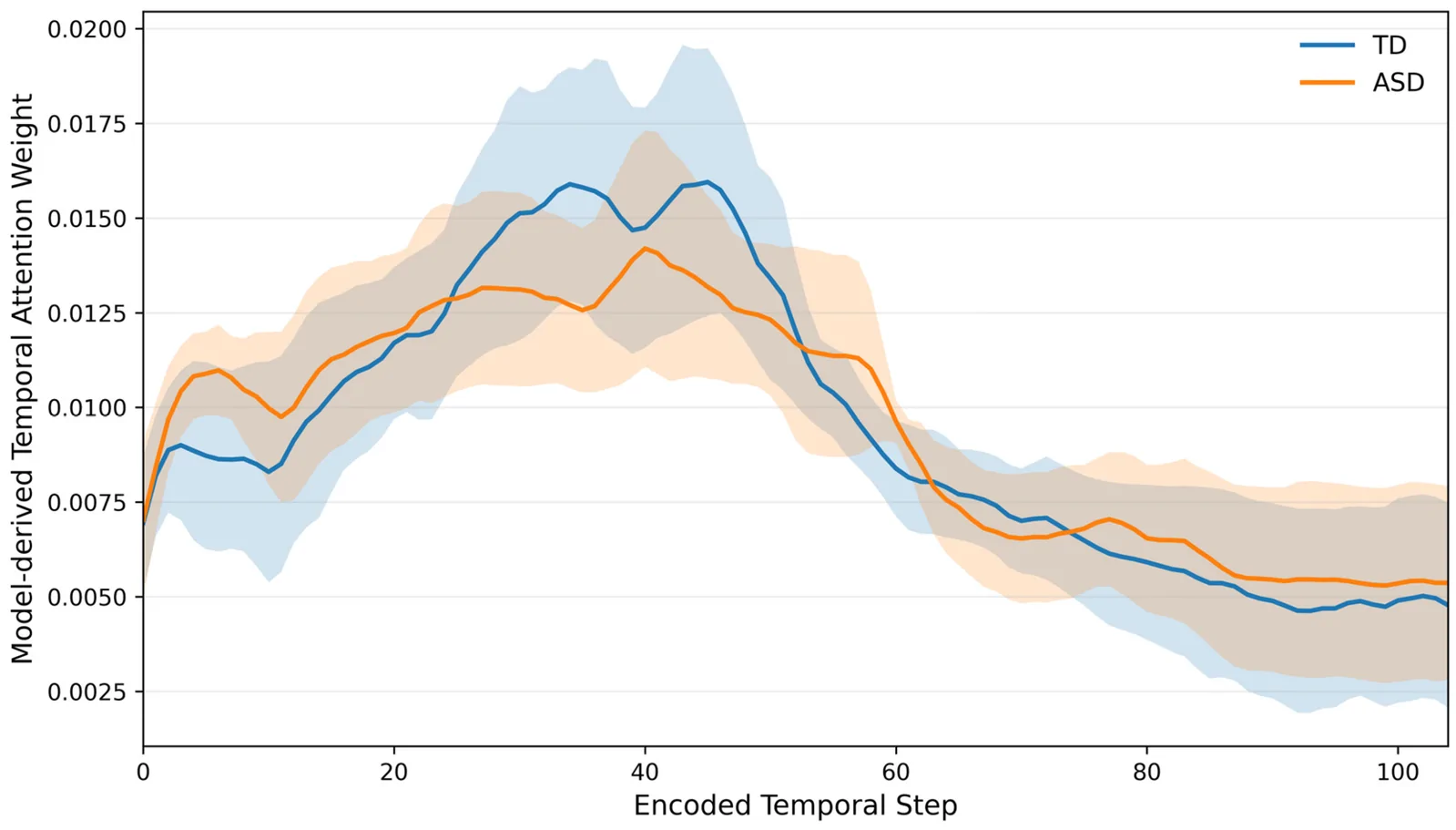
Model-derived temporal attention profiles of dynamic gaze sequences.

The model-derived temporal attention weights of the dynamic sequences are shown in Figure 4. Raw gaze sequences were aligned to 211 sampling points and encoded by the temporal feature extraction network into 105 consecutive hidden temporal steps (Step_0–Step_104). Because each sequence corresponded to an individual cue stage defined by recorded cue timestamps, these steps represent relative temporal positions within each gaze sequence rather than stages of the complete trial. For each participant-level split, attention weights at each encoded step were first averaged across all valid gaze segments of the same test participant, yielding one 105-step profile per participant. These profiles were then averaged separately for the ASD and TD groups and summarized across the five random splits. The solid lines represent the mean profiles across the five splits, and the shaded bands indicate the corresponding standard deviations.

The model-derived attention profiles showed different descriptive distributions between groups. The TD group showed relatively higher mean weights around the middle of the encoded sequence, peaking at approximately Step 45, whereas the ASD group showed a broader pattern with relatively elevated weights across several early and middle positions. These results indicate that the trained models assigned different relative weights to encoded temporal positions within the gaze segments. However, because shorter sequences were zero-padded and longer sequences were truncated, patterns at the early and particularly late positions should be interpreted cautiously.

#### 3.8.3. Feature Selection Stability and Conditional Gradient-Based Saliency

**Figure 5:**
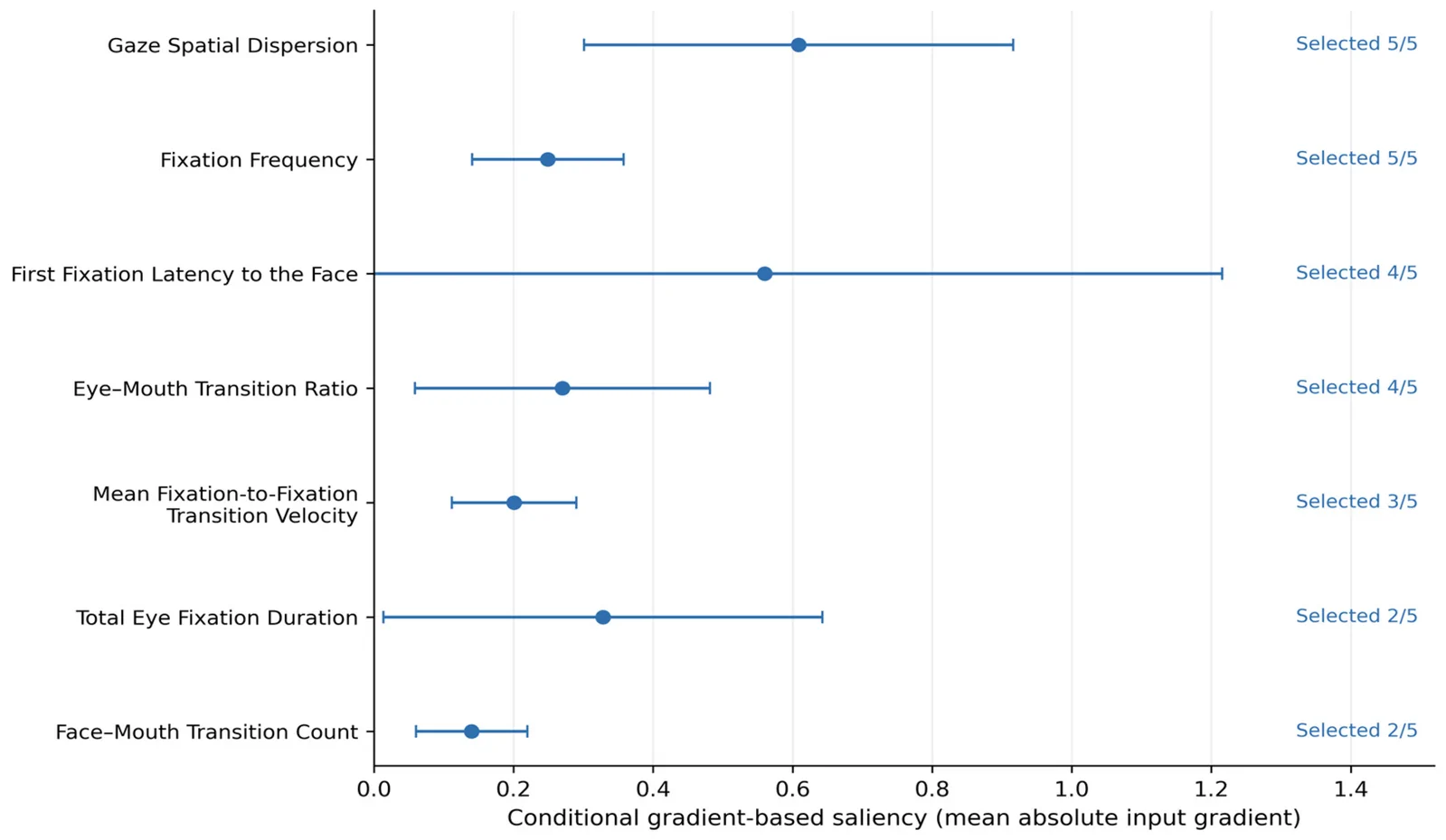
Static feature selection frequency and conditional gradient-based saliency across five repeated participant-level evaluations.

To further characterize the static feature selection results, selection frequency and conditional gradient-based saliency were summarized across the five repeated participant-level evaluations for the seven most frequently selected features. As shown in Figure 5, the labels on the right indicate the number of evaluations in which each feature was selected, while the points and horizontal error bars represent the mean conditional gradient-based saliency and its variability across the evaluations in which the corresponding feature was selected.

Gaze Spatial Dispersion and Fixation Frequency were selected in all five evaluations, with mean conditional gradient-based saliency values of 0.609 ± 0.308 and 0.249 ± 0.109, respectively. First Fixation Latency to the Face and Eye–Mouth Transition Ratio were each selected in four of the five evaluations, with corresponding mean saliency values of 0.560 ± 0.656 and 0.270 ± 0.211. Mean Fixation-to-Fixation Transition Velocity was selected in three evaluations, with a mean saliency value of 0.201 ± 0.089, while Total Eye Fixation Duration and Face–Mouth Transition Count were each selected in two evaluations.

For conditional gradient-based saliency, Gaze Spatial Dispersion and First Fixation Latency to the Face showed relatively higher mean values, although the latter also showed greater variability across evaluations. Fixation Frequency showed a comparatively lower mean saliency value despite being selected in all five evaluations. The remaining features showed lower mean conditional saliency values with varying degrees of variability across evaluations.

### 3.9. Exploratory Analyses

In addition to the primary analyses and the fully nested LOPO evaluation reported above, several supplementary analyses were retained to provide further information on model performance and sensitivity. These analyses used the exploratory static feature specification established from the full dataset before repeated participant-level model evaluation and may therefore be subject to optimistic bias. Accordingly, the results in this section are presented as exploratory findings and are not used as the primary evidence for classification performance.

Under the exploratory feature specification, additional comparisons were conducted across different feature settings and network architectures, with the complete results provided in Supplementary Table S6. To illustrate the performance pattern associated with different feature integration strategies within the same network architecture, CNN-LSTM was used as the representative model. With dynamic sequences alone, CNN-LSTM achieved an accuracy of 0.78 ± 0.11, ASD recall of 0.65 ± 0.29, F1-score of 0.69 ± 0.21, and AUC of 0.84 ± 0.09. After static gaze features were introduced through early feature concatenation, the corresponding values were 0.78 ± 0.15, 0.65 ± 0.28, 0.70 ± 0.23, and 0.87 ± 0.10, respectively. With dynamic and static multiscale feature fusion, CNN-LSTM achieved an accuracy of 0.80 ± 0.10, ASD recall of 0.80 ± 0.21, F1-score of 0.78 ± 0.08, and AUC of 0.89 ± 0.10.

Descriptively, early feature concatenation produced only limited changes relative to dynamic sequences alone, whereas multiscale fusion showed higher mean values across the four reported metrics. Given the exploratory feature specification used in these analyses, these differences are reported as descriptive patterns rather than as primary performance results.

An exploratory ablation analysis was further conducted for the behavioral semantic branch. As shown in Table 10, the full CNN-LSTM multiscale fusion model achieved an accuracy of 0.80 ± 0.10, ASD recall of 0.80 ± 0.21, F1-score of 0.78 ± 0.08, and AUC of 0.89 ± 0.10. After removal of the behavioral semantic branch, the corresponding values were 0.78 ± 0.10, 0.70 ± 0.27, 0.72 ± 0.19, and 0.86 ± 0.13, respectively. Lower mean values were therefore observed across all four metrics after removal of the behavioral semantic branch.

A sequence-length sensitivity analysis was also conducted by reducing the maximum sequence length from 211 to 165 sampling points, corresponding to approximately 5 s of gaze data. Under the 165-point setting, the CNN-LSTM multiscale fusion model achieved an accuracy of 0.80 ± 0.14, ASD recall of 0.80 ± 0.11, F1-score of 0.79 ± 0.11, and AUC of 0.89 ± 0.11. These values were descriptively similar to those obtained under the 211-point setting.

## 4. Discussion

### 4.1. Discriminative Value of Static Statistical Features

Static statistical gaze features retained participant-level discriminative information, although performance varied across classifiers and participant-level evaluations. RF achieved the highest mean accuracy of 0.76 ± 0.09, whereas SVM achieved the highest mean AUC of 0.85 ± 0.20 and ASD recall of 0.70 ± 0.33. The relatively large variability in several metrics, particularly ASD recall, indicates that classification performance remained sensitive to participant composition across evaluations. This variability should be considered when interpreting static-feature performance in the present small-sample setting. Previous studies have similarly reported substantial individual variability in gaze-based characteristics associated with ASD [30].

The feature-selection results further showed that the retained static feature set was not identical across participant-level evaluations. Gaze Spatial Dispersion and Fixation Frequency were selected in all five evaluations, while First Fixation Latency to the Face and Eye-Mouth Transition Ratio were each selected in four of the five evaluations. Other features were retained less consistently. Rather than supporting a single fixed set of universally discriminative gaze measures, this pattern suggests that the static features retained by the selection procedure were partly dependent on the composition of the training participants. Nevertheless, the repeated selection of Gaze Spatial Dispersion and Fixation Frequency indicates that information related to overall gaze distribution and fixation activity was retained relatively consistently across the five evaluations. These observations are broadly consistent with previous studies reporting differences in visual attention allocation and gaze behavior during social viewing contexts [31,32].

Conditional gradient-based saliency provided complementary model-level information about the selected static features. Gaze Spatial Dispersion showed both consistent selection across all five evaluations and relatively high conditional saliency. First Fixation Latency to the Face also showed relatively high mean saliency but greater variability across evaluations. In contrast, Fixation Frequency was selected in all five evaluations despite showing a comparatively lower mean saliency value. These patterns indicate that selection frequency and conditional gradient-based saliency characterize different aspects of the fitted models. Selection frequency reflects how consistently a feature was retained across participant-level evaluations, whereas conditional saliency reflects model sensitivity to that feature in the evaluations in which it was selected. A feature that is consistently retained therefore does not necessarily show the highest model sensitivity, while a feature with relatively high saliency may still show substantial variability across participant splits.

These model-based analyses should not be interpreted as direct evidence of the behavioral, cognitive, or biological importance of individual gaze measures. Gradient-based saliency reflects the sensitivity of the fitted classifier to changes in its input rather than causal contribution or explained variance. The observed feature-selection and saliency patterns should therefore be regarded as descriptive characteristics of the fitted models within the present sample.

The exploratory analyses based on the fixed seven-feature specification derived from the full dataset provided additional descriptive information on static gaze representations. However, because this feature specification was established using the full dataset, these exploratory findings are considered supplementary to the primary participant-level analyses rather than evidence for a fixed set of predictive static gaze features.

### 4.2. Characterization of Temporal Gaze Features Using Dynamic Sequences

Dynamic sequences provided participant-level discriminative information while preserving the temporal progression of gaze behavior. CNN-LSTM achieved an accuracy of 0.78 ± 0.11 and an AUC of 0.84 ± 0.09, whereas TCN achieved the highest ASD recall of 0.75 ± 0.18 among the evaluated dynamic sequence models. Rather than showing a uniform advantage over static statistical features, these results indicate that dynamic sequences provided an alternative representation of gaze information with comparable participant-level classification performance. Previous studies have shown that scanpath and similarity features can reflect differences in visual exploration among children with ASD during emotional face processing, indicating that dynamic sequences contain discriminative information [33]. Spatiotemporal gaze analysis has also indicated that children with ASD show reduced face prioritization during early social visual processing, which is associated with adaptive functioning, face recognition, and symptom severity, supporting the behavioral relevance of dynamic gaze patterns [34]. However, performance varied across sequence architectures, with BiLSTM showing lower mean accuracy and AUC than CNN, TCN, and CNN-LSTM.

This variation may partly reflect the difficulty of learning generalizable long-range dependencies from small and noisy samples using recurrent-only models. Gaze sequences contain not only temporal dependencies but also local changes in gaze position and ROI allocation, and relying on a single sequence model may be insufficient to fully capture these characteristics [35]. Measurement noise and individual variability in gaze behavior may further contribute to differences in performance across sequence models [36].

The temporal attention analysis provided additional model-level information on how sequential gaze information was utilized. The learned attention profiles were distributed across the sequence rather than concentrated uniformly at all temporal positions, indicating that the fitted model assigned different weights to different parts of the observed gaze sequence. However, these attention weights do not establish that particular temporal positions correspond to distinct cognitive or behavioral processing stages. They should instead be interpreted as internal weighting patterns of the fitted model within the present sample.

Taken together, the static and dynamic results suggest that the two representations characterize different aspects of gaze behavior. Static statistical features summarize overall viewing characteristics, whereas dynamic sequences preserve changes in gaze position and ROI allocation over time. Their similar participant-level classification performance does not establish that one representation is generally superior to the other, but provides a rationale for examining how information from the two representations can be integrated. The temporal localization of the model-derived patterns should also be interpreted cautiously because fixed-length preprocessing, including zero-padding and truncation, may affect the representation of temporal information.

### 4.3. Effects of Dynamic and Static Feature Integration Strategies

An important finding of the present study is that introducing additional static gaze information did not automatically improve classification performance. Early feature concatenation produced heterogeneous changes across network architectures. CNN and CNN-LSTM showed decreases in mean accuracy and ASD recall after early concatenation, whereas BiLSTM showed improvements in some metrics and TCN showed lower mean values across all four metrics. These results indicate that simply adding static information to dynamic representations through direct concatenation did not produce a consistent improvement across architectures.

In comparison with early feature concatenation, multiscale fusion showed a more favorable descriptive pattern. Across all four network architectures, multiscale fusion achieved higher mean accuracy, F1-score, and AUC, while ASD recall was also higher for CNN and CNN-LSTM and remained unchanged for BiLSTM and TCN. Although the magnitude of these differences varied across architectures and evaluation metrics, the overall pattern suggests that multiscale fusion provided a more consistent way of combining static and dynamic gaze information than direct early concatenation. These differences should nevertheless be interpreted descriptively rather than as evidence of a general performance advantage.

The different performance patterns of early concatenation and multiscale fusion may be related to how heterogeneous gaze information is represented before integration. Direct concatenation combines static and dynamic information at an early stage despite differences in their statistical and temporal properties. In contrast, the multiscale framework represents different sources of gaze information separately before integrating them within a unified representation. This organization may allow heterogeneous information to be incorporated without relying solely on direct feature stacking. Previous studies have shown that complementary features can support the modeling of complex visual attention [37]. Multilevel representations may also facilitate the representation of heterogeneous information under small-sample conditions [38], while feature organization and integration strategies may influence model performance [39].

Comparison with the dynamic sequence models further showed that the effect of multiscale fusion varied across network architectures. BiLSTM showed the clearest overall improvement, with higher mean accuracy, F1-score, and AUC after multiscale fusion, although ASD recall was slightly lower. CNN and TCN showed improvements in some metrics but not others, while CNN-LSTM showed broadly comparable mean performance between multiscale fusion and dynamic sequences alone. These architecture-dependent patterns suggest that multiscale fusion can provide additional benefits for some sequence representations, but the effect is not uniform across models or evaluation metrics. Overall, the findings highlight that the value of heterogeneous gaze information depends not only on whether additional features are introduced, but also on how they are represented and integrated within the model.

### 4.4. Robustness and Model Interpretation Analyses

The fully nested LOPO evaluation provided additional support for the participant-level classification results under a stricter internal validation procedure. Participant-level discrimination remained observable when each held-out participant was completely excluded from static feature selection and model development, with an AUC of 0.87 and an ASD recall of 0.78. This result is particularly relevant given the variability in static feature selection observed across the repeated participant-level evaluations. It suggests that the classification results were not dependent on allowing information from the held-out participant to influence the selection of static features. Nevertheless, the LOPO analysis remains an internal validation within the same cohort and should not be interpreted as evidence of external generalizability.

The gating and attention analyses further showed that the framework utilized information from multiple levels of gaze representation rather than relying on a single source. The dynamic–static fusion and behavioral semantic representations received relatively greater average gating weights, while the ROI transition representation received a lower weight. This pattern is consistent with the different branches playing differentiated roles within the framework. Dynamic and static features provide information about temporal gaze changes and overall viewing characteristics, whereas ROI transitions and behavioral semantic representations describe gaze behavior from spatial and higher-level perspectives. The different weighting patterns therefore support the use of a multilevel representation in which heterogeneous gaze information is organized according to its characteristics before integration. These model-derived weights should be interpreted as indicators of how information was organized within the framework rather than as direct evidence of behavioral or causal importance.

The exploratory ablation analysis provided complementary evidence for this interpretation. Removing the behavioral semantic branch was accompanied by lower mean performance across the evaluated metrics, with more apparent changes in ASD recall and F1-score. Together with the gating results, this pattern suggests that the behavioral semantic representation contributed information that was not fully captured by the dynamic–static and ROI transition representations alone. Rather than replacing the lower-level gaze features, the semantic branch may therefore serve as a complementary representation that organizes gaze patterns at a higher level.

The sequence-length sensitivity analysis provided an additional perspective on the temporal representation. The broadly similar performance obtained with the shorter sequence length suggests that the exploratory classification pattern was not strongly dependent on the original maximum sequence length. At the same time, the analysis does not eliminate the possible influence of truncation and zero-padding on temporal information. These findings further illustrate the value of organizing heterogeneous gaze information at different representational levels before integration, which is consistent with the design of the multiscale framework.

### 4.5. Comparison with Previous Studies and Study Implications

Compared with previous studies, the classification performance observed in the present study was within the range reported by previous eye-tracking studies using participant-independent evaluation [15,28,29]. However, direct performance comparisons across studies should be made cautiously because of differences in participant characteristics, experimental tasks, eye-tracking systems, feature representations, and validation procedures. Participant-independent evaluation separates participants between model development and testing and therefore provides a more appropriate assessment of participant-level classification than trial-level random partitioning. The participant-level results obtained in the present study indicate that discriminative information remained observable when evaluation was conducted on participants who were not included in model training.

Previous studies have suggested that combining complementary representations can provide different sources of information for classification [23]. Multilevel feature representations may further support the organization of heterogeneous information [24], while fusion strategies can influence how information from different representations is utilized by a model [40]. The present findings are broadly consistent with this perspective. In particular, the different performance patterns observed for early concatenation and multiscale fusion indicate that the value of combining gaze representations may depend on how heterogeneous information is represented and integrated rather than simply on the inclusion of additional features.

The proposed framework therefore provides a way to examine different aspects of gaze information within a unified modeling framework. Static statistical features characterize overall viewing patterns, dynamic sequences preserve temporal changes in gaze behavior, and the additional representations describe ROI transitions and higher-level behavioral patterns. Organizing these representations at different levels may help characterize how heterogeneous gaze information is utilized during participant classification and provide a basis for further investigation of gaze patterns associated with ASD.

## 5. Conclusions and Limitations

### 5.1. Conclusions

This study investigated participant classification using eye-tracking data collected from children with pre-existing clinical ASD diagnoses and TD children aged 4–7 years during an emotion recognition task. Static statistical features, dynamic gaze sequences, early feature concatenation, and dynamic and static multiscale feature fusion were systematically evaluated under participant-level validation. The results showed that both static and dynamic gaze representations contained information relevant to participant classification, while their performance varied across models and evaluation settings.

Early feature concatenation did not consistently improve classification performance. In comparison, multiscale fusion showed a more favorable descriptive pattern than early concatenation across the evaluated network architectures. However, its performance relative to dynamic sequences alone varied across models and evaluation metrics. These findings suggest that different approaches to representing and combining gaze features may lead to different classification outcomes, and the observed advantages of multiscale fusion should therefore be interpreted as descriptive rather than generalizable performance improvements.

The fully nested LOPO evaluation provided additional internal validation of the participant-level classification results. Overall, this study provides a systematic comparison of different gaze representations and feature integration approaches for participant classification. The proposed multiscale framework offers one approach for combining static and dynamic gaze representations, but its relative performance varied across model architectures and evaluation metrics. Further studies using larger independent samples, different tasks, and external validation are needed to determine the robustness and generalizability of these findings.

### 5.2. Limitations

The present study has several limitations. The effective sample size was relatively small, which may limit the stability of performance estimates and the ability of deep learning models to learn complex temporal patterns. Performance estimates may therefore be sensitive to participant variation across data splits. Although the fully nested LOPO evaluation provided additional internal validation, it was conducted within the same cohort and does not constitute independent external validation. Future studies should include larger samples and independent external datasets.

Several methodological issues should also be considered. Although static feature selection was restricted to the training participants in the primary analyses, the selected features varied across participant-level evaluations, which may partly reflect the limited sample size. The fixed seven-feature specification derived from the full dataset was used only in the exploratory analyses, and the corresponding findings should therefore be interpreted cautiously because of potential optimistic bias. The relatively low gaze-sampling frequency of 33 Hz limits the temporal resolution for characterizing rapid eye movements, and velocity-related measures should therefore be interpreted as coarse descriptors of overall gaze movement rather than precise estimates of saccadic dynamics. The dynamic sequence models used fixed-length zero-padding without explicit temporal masking, and group differences in the proportion of valid gaze samples within retained segments may represent a potential source of bias. The maximum sequence length of 211 sampling points may also have resulted in the loss of later viewing behavior through truncation, although the sensitivity analysis using 165 sampling points showed a broadly consistent performance pattern. Thus, the possibility that the models partially captured information related to padding or data quality cannot be excluded. Future studies should consider explicit temporal masking or length-aware modeling, more rigorous missing-data handling, a broader range of sequence lengths, and higher-frequency eye-tracking devices. Variation in the number of valid trials across participants may also have affected the stability of participant-level probability estimates and should be further evaluated using standardized trial counts.

The current data were collected from a single emotion recognition task, and the applicability of the framework to other social cognition contexts, such as free viewing, dynamic videos, and real-world interactions, remains to be examined. Because graded cues were response-contingent, differences in cue exposure and the number of gaze segments across participants may also have introduced task-related variability. Future studies should evaluate gaze representations across broader task paradigms and independent samples, while more systematically recording and analyzing task-exposure variables such as reaction time, cue number, trial or stage duration, and cumulative visual exposure, and incorporating these variables into sensitivity analyses where appropriate.

## Figures and Tables

**Table 1:** Demographic characteristics of children in the ASD and TD groups.

Variable	ASD Group (*n* = 18)	TD Group (*n* = 26)	Statistic	*p*-Value
Gender, male/female (*n*)	12/6	13/13	χ^2^ = 1.22	0.269
Age (years, M ± SD)	5.50 ± 0.80	5.23 ± 0.65	t = 1.22	0.228

**Table 2:** Performance of models using static statistical features.

Model	Accuracy	ASD Recall	F1-Score	AUC
KNN	0.73 ± 0.13	0.65 ± 0.34	0.65 ± 0.22	0.81 ± 0.17
LR	0.73 ± 0.15	0.60 ± 0.38	0.62 ± 0.26	0.82 ± 0.19
RF	0.76 ± 0.09	0.65 ± 0.34	0.67 ± 0.19	0.83 ± 0.22
SVM	0.73 ± 0.18	0.70 ± 0.33	0.67 ± 0.24	0.85 ± 0.20
XGBoost	0.69 ± 0.12	0.55 ± 0.27	0.59 ± 0.18	0.78 ± 0.21

**Table 3:** Performance of models using dynamic sequences.

Model	Accuracy	ASD Recall	F1-Score	AUC
BiLSTM	0.62 ± 0.10	0.70 ± 0.21	0.62 ± 0.11	0.55 ± 0.15
CNN	0.76 ± 0.09	0.70 ± 0.21	0.71 ± 0.11	0.82 ± 0.10
TCN	0.71 ± 0.13	0.75 ± 0.18	0.69 ± 0.14	0.82 ± 0.09
CNN-LSTM	0.78 ± 0.11	0.65 ± 0.29	0.69 ± 0.21	0.84 ± 0.09

**Table 4:** Performance of models using early feature concatenation.

Model	Accuracy	ASD Recall	F1-Score	AUC
BiLSTM	0.71 ± 0.13	0.65 ± 0.22	0.66 ± 0.16	0.77 ± 0.18
CNN	0.71 ± 0.15	0.60 ± 0.29	0.63 ± 0.21	0.71 ± 0.18
TCN	0.69 ± 0.18	0.60 ± 0.28	0.61 ± 0.25	0.73 ± 0.14
CNN-LSTM	0.76 ± 0.15	0.60 ± 0.29	0.66 ± 0.22	0.75 ± 0.15

**Table 5:** Performance of models using dynamic and static multiscale feature fusion.

Model	Accuracy	ASD Recall	F1-Score	AUC
BiLSTM	0.76 ± 0.18	0.65 ± 0.22	0.71 ± 0.18	0.81 ± 0.16
CNN	0.78 ± 0.11	0.70 ± 0.28	0.71 ± 0.16	0.73 ± 0.19
TCN	0.75 ± 0.15	0.60 ± 0.28	0.66 ± 0.22	0.75 ± 0.15
CNN-LSTM	0.78 ± 0.08	0.65 ± 0.22	0.71 ± 0.12	0.76 ± 0.16

**Table 6:** Comparison of multiscale fusion and early concatenation.

Model	Accuracy	ASD Recall	F1-Score	AUC
BiLSTM	+0.05	+0.00	+0.05	+0.04
CNN	+0.07	+0.10	+0.08	+0.02
TCN	+0.06	+0.00	+0.05	+0.02
CNN-LSTM	+0.02	+0.05	+0.05	+0.01

**Table 7:** Performance of the fully nested LOPO evaluation.

Accuracy	ASD Recall	F1-Score	AUC	Specificity	Precision	NPV	Brier Score
0.75	0.78	0.72	0.87	0.73	0.67	0.83	0.163

**Table 8:** Comparison of representative models across the four feature settings.

Feature Setting	Representative Model	Accuracy	ASD Recall	F1-Score	AUC
Static statistical features	RF	0.76 ± 0.09	0.65 ± 0.34	0.67 ± 0.19	0.83 ± 0.22
Dynamic sequences	CNN-LSTM	0.78 ± 0.11	0.65 ± 0.29	0.69 ± 0.21	0.84 ± 0.09
Early feature concatenation	CNN-LSTM	0.76 ± 0.15	0.60 ± 0.29	0.66 ± 0.22	0.75 ± 0.15
Dynamic and static multiscale feature fusion	CNN-LSTM	0.78 ± 0.08	0.65 ± 0.22	0.71 ± 0.12	0.76 ± 0.16

**Table 9:** Summary of previous studies and the present study.

Studies	*n*	The Type of Feature	Model	Validation	Accuracy
Cilia et al. (2021) [15]	59	scanpaths	CNN	Participant- level 3-fold	0.71
Iwauchi et al. (2023) [28]	30	Statistical Features	Weighted CNN	LOPO-CV	0.667
Hudry et al. (2025) [29]	196	Statistical Features	Gazefinder classification algorithm	Participant- level LOOCV	0.75
This study	44	dynamic and static multiscale feature fusion	CNN-LSTM	Participant- level random holdout	0.78 ± 0.08
This study	44	early feature concatenation	CNN-LSTM	Participant- level random holdout	0.76 ± 0.15
This study	44	dynamic sequences	CNN-LSTM	Participant- level random holdout	0.78 ± 0.11
This study	44	static statistical features	RF	Participant- level random holdout	0.76 ± 0.09

**Table 10:** Exploratory ablation analysis of the behavioral semantic branch.

Model	Accuracy	ASD Recall	F1-Score	AUC
Full CNN-LSTM multiscale fusion model	0.80 ± 0.10	0.80 ± 0.21	0.78 ± 0.08	0.89 ± 0.10
Without behavioral semantic branch	0.78 ± 0.10	0.70 ± 0.27	0.72 ± 0.19	0.86 ± 0.13

## Data Availability

The experimental data generated during the study are not publicly available due to privacy and ethical restrictions.

## Supplementary Materials

The following supporting information can be downloaded at https://www.mdpi.com/article/10.3390/jemr19050111/s1. Tables S1–S12 provide split-specific static features selected using training participants only; definitions of the fixed seven static features used in the exploratory analyses; paired performance differences between multiscale fusion and early feature concatenation, with corresponding 95% confidence intervals, in the primary analyses; approximate descriptive 95% confidence intervals for model performance metrics in the primary analyses; a descriptive comparison with previous eye-tracking studies; exploratory model results and participant-level classification details; approximate descriptive 95% confidence intervals for model performance metrics in the exploratory analyses; paired performance differences with corresponding 95% confidence intervals in the exploratory analyses; participant-level gaze-data quality information; split-level classification results under training-only static feature selection; and participant-level confusion matrices and additional classification metrics for the CNN–LSTM multiscale fusion model in the primary analyses.

## Abbreviations

The following abbreviations are used in this manuscript:

ASD	Autism Spectrum Disorder
TD	Typically Developing
CNN	Convolutional Neural Network
BiLSTM	Bidirectional Long Short-Term Memory
TCN	Temporal Convolutional Network
CNN-LSTM	Convolutional Neural Network–Long Short-Term Memory
SVM	Support Vector Machine
RF	Random Forest
LR	Logistic Regression
XGBoost	Extreme Gradient Boosting
KNN	K-Nearest Neighbors
ROI	Region of Interest
AUC	Area Under the Curve
ADI-R	Autism Diagnostic Interview-Revised
ADOS-G	Autism Diagnostic Observation Schedule-Generic
