# Supplementary material for: Exploring ASD Identification Using Different Types of Eye-Tracking Features
Source: J Eye Mov Res. 2026 Oct 2;19(5):111. doi: 10.3390/jemr19050111 (PMC13706125; doi:10.3390/jemr19050111)
Supplement: Supplementary file 1 [file jemr-19-00111-s001.zip › jemr-4508310-supplementary.pdf]

Supplementary Materials

1. **Supplementary Table S1**. Static features selected across the five repeated participant-level evaluations.

Split	Selected static features
1	Gaze Spatial Dispersion; Fixation Frequency; Eye–Mouth Transition Ratio; First Fixation Latency to the Face; Mean Fixation-to-Fixation Transition Velocity; Mean Distance Between Fixations; Average Scanpath Velocity
2	Gaze Spatial Dispersion; Fixation Frequency; Eye–Mouth Transition Ratio; First Fixation Latency to the Face; Total Eye Fixation Duration; Mean Fixation-to-Fixation Transition Velocity; Scanpath Length
3	Gaze Spatial Dispersion; Fixation Frequency; Face–Mouth Transition Count; Eye–Mouth Transition Ratio; Mean Fixation-to-Fixation Transition Velocity; Total Gaze Shifts; Mean Face Fixation-to-Fixation Transition Interval
4	Fixation Frequency; Gaze Spatial Dispersion; First Fixation Latency to the Face; Total Eye Fixation Duration; Eye–Mouth Transition Ratio; Mean Eye Fixation Duration; Mean Face Fixation Duration
5	Fixation Frequency; Gaze Spatial Dispersion; Median Fixation Duration; Mean Eye Fixation-to-Fixation Transition Distance; Face–Mouth Transition Count; First Fixation Latency to the Face; Median Face Fixation Duration

Note. Seven features were retained in each split. The transition-related candidate features were calculated from detected fixations. For two successive fixations, the transition interval was defined as the time between the end of the preceding fixation and the onset of the subsequent fixation, the transition distance was calculated as the Euclidean distance between their mean gaze coordinates in pixels, and the transition velocity was calculated as the transition distance divided by the corresponding interval in seconds. For AOI-specific measures, fixations assigned to the corresponding AOI were first extracted, and transition measures were then calculated between successive fixations in the AOI-specific fixation sequence. Accordingly, these measures are reported as Mean Fixation-to-Fixation Transition Velocity, Mean Face Fixation-to-Fixation Transition Interval, and Mean Eye Fixation-to-Fixation Transition Distance to reflect their operational definitions. Given the 33 Hz sampling rate and the absence of an independent saccade detector, these measures were interpreted as coarse descriptors of fixation-to-fixation gaze transitions rather than precise measures of saccadic kinematics.

2. **Supplementary Table S2.** Operational definitions of the fixed seven static eye-tracking features used in the **exploratory analyses**.

Feature	Operational definition
Gaze Spatial Dispersion	SD of horizontal normalized gaze coordinates.
Fixation Frequency	Number of detected fixations per second.
Total Face Fixation Duration	Total fixation duration within the face ROI.

Feature	Operational definition
Eye-Mouth Transition Ratio	Proportion of bidirectional Eye–Mouth transitions among all ROI transitions.
First Fixation Latency to the Face	Time from trial onset to the first fixation within the face ROI.
Average Scanpath Velocity	Total gaze-path displacement divided by trial duration (pixels/s), reflecting overall gaze movement rather than saccadic peak velocity.
Mean Eye Fixation Duration	Mean fixation duration within the eye ROI.

Note. These seven static features constitute the fixed feature specification used only in the exploratory analyses.

3. **Supplementary Table S3.** Paired performance differences and 95% confidence intervals between multiscale fusion and early feature concatenation in the primary analyses across the five matched participant-level splits.

Model	Accuracy [95% CI]	ASD Recall [95% CI]	F1-Score [95% CI]	AUC [95% CI]
BiLSTM	+0.05 [−0.08, 0.17]	+0.00 [0.00, 0.00]	+0.05 [−0.04, 0.13]	+0.04 [−0.03, 0.11]
CNN	+0.07 [−0.01, 0.14]	+0.10 [−0.07, 0.27]	+0.08 [−0.03, 0.20]	+0.02 [−0.12, 0.16]
TCN	+0.06 [−0.06, 0.19]	+0.00 [0.00, 0.00]	+0.05 [−0.04, 0.14]	+0.02 [−0.04, 0.08]
CNN–LSTM	+0.02 [−0.13, 0.17]	+0.05 [−0.09, 0.19]	+0.05 [−0.09, 0.19]	+0.01 [−0.11, 0.13]

Note. Δ represents the mean performance difference between multiscale fusion and early feature concatenation (multiscale fusion − early feature concatenation). Values in brackets indicate 95% confidence intervals for the paired differences across the five matched participant-level splits. Two-sided Wilcoxon signed-rank tests were used for paired statistical comparisons across the five matched participant-level splits; none of the comparisons reached statistical significance (all $p > 0.05$).

4. **Supplementary Table S4.** Approximate descriptive 95% confidence intervals for model performance metrics from the primary analyses across four feature settings and five repeated participant-level evaluations.

Feature setting	Model	Accuracy 95% CI	ASD Recall 95% CI	F1-score 95% CI	AUC 95% CI
Static statistical features	KNN	[0.54, 0.86]	[0.23, 0.92]	[0.36, 0.86]	[0.52, 0.94]
	LR	[0.51, 0.87]	[0.17, 0.91]	[0.29, 0.87]	[0.48, 0.96]
	RF	[0.63, 0.85]	[0.23, 0.92]	[0.41, 0.86]	[0.41, 0.97]
	SVM	[0.47, 0.89]	[0.25, 0.94]	[0.35, 0.89]	[0.45, 0.98]
	XGBoost	[0.53, 0.82]	[0.24, 0.83]	[0.36, 0.78]	[0.44, 0.94]
Dynamic sequences	BiLSTM	[0.49, 0.73]	[0.40, 0.89]	[0.48, 0.74]	[0.37, 0.72]
	CNN	[0.63, 0.85]	[0.40, 0.89]	[0.56, 0.83]	[0.66, 0.91]
	TCN	[0.53, 0.84]	[0.48, 0.91]	[0.50, 0.83]	[0.68, 0.91]
	CNN–LSTM	[0.62, 0.89]	[0.28, 0.90]	[0.40, 0.88]	[0.70, 0.92]
Early feature concatenation	BiLSTM	[0.53, 0.84]	[0.36, 0.86]	[0.44, 0.82]	[0.49, 0.92]
	CNN	[0.50, 0.86]	[0.25, 0.87]	[0.36, 0.84]	[0.45, 0.88]
	TCN	[0.44, 0.86]	[0.25, 0.87]	[0.30, 0.85]	[0.53, 0.87]
	CNN–LSTM	[0.53, 0.90]	[0.25, 0.87]	[0.36, 0.87]	[0.53, 0.89]
Dynamic and static multiscale fusion	BiLSTM	[0.48, 0.92]	[0.36, 0.86]	[0.45, 0.88]	[0.54, 0.94]
	CNN	[0.62, 0.89]	[0.31, 0.92]	[0.48, 0.87]	[0.45, 0.90]
	TCN	[0.53, 0.89]	[0.26, 0.86]	[0.36, 0.87]	[0.53, 0.89]
	CNN–LSTM	[0.67, 0.86]	[0.36, 0.86]	[0.54, 0.83]	[0.52, 0.90]

Note. Values represent approximate descriptive 95% confidence intervals for performance metrics across the five repeated participant-level evaluations.

5. **Supplementary Table S5.** Comparison of previous eye tracking studies on ASD classification with the present study.

Studies	n / Age	Task	Eye tracker	Diagnosis	Feature / Model	Validation	Metrics
Cilia et al.（2021）[15]	59 / ~8 y	Social photo/video viewing	SMI RED250, 250 Hz	ADI-R, ADOS-G	Scanpaths / CNN	Participant-level 3-fold CV	Acc ≈ 0.71
Iwauchi et al.（2023）[28]	30/ ~10–11 y	FEIT	Tobii Pro Fusion, 120 Hz	DSM-5, ADOS-2	Statistical features / Weighted CNN	LOPO-CV	Acc=0.667; Sens.=0.600; Spec.=0.733
Hudry et al.（2025）[29]	196 / 24–49 m	Gazefinder assessment	Gazefinder, 50 Hz	Community diagnosis	Statistical features / Gazefinder algorithm	Participant-level LOOCV	Acc=0.75; AUC=0.82; Sens.=0.81; Spec.=0.69
This study	44 / 4–7 y	Facial emotion recognition	Tobii Eye Tracker 5, 33 Hz	Hospital/institutional clinical diagnosis	Multiscale fusion / CNN–LSTM	Participant-level random holdout	Acc = 0.78± 0.08; ASD Recall = 0.65 ± 0.22; F1 = 0.71 ± 0.12; AUC = 0.76 ± 0.16
This study	44 / 4–7 y	Facial emotion recognition	Tobii Eye Tracker 5, 33 Hz	Hospital/institutional clinical diagnosis	early feature concatenation/CNN-LSTM	Participant-level random holdout	Acc = 0.76 ± 0.15; ASD Recall = 0.60 ± 0.29; F1 = 0.66 ± 0.22; AUC = 0.75 ± 0.15
This study	44 / 4–7 y	Facial emotion recognition	Tobii Eye Tracker 5, 33 Hz	Hospital/institutional clinical diagnosis	dynamic sequences/CNN-LSTM	Participant-level random holdout	Acc = 0.78 ± 0.11; ASD Recall = 0.65 ± 0.29; F1 = 0.69 ± 0.21; AUC = 0.84 ± 0.09
This study	44 / 4–7 y	Facial emotion recognition	Tobii Eye Tracker 5, 33 Hz	Hospital/institutional clinical diagnosis	static statistical features/RF	Participant-level random holdout	Acc = 0.76 ± 0.09; ASD Recall = 0.65 ± 0.34; F1 = 0.67 ± 0.19; AUC = 0.83 ± 0.22

Note. The studies differ in participant characteristics, experimental tasks, eye tracking devices, diagnostic procedures, feature representations, and validation strategies. Therefore, the reported performance metrics are presented for descriptive comparison only and should not be interpreted as direct evidence of performance superiority across studies. Acc, accuracy; Sens., sensitivity; Spec., specificity; AUC, area under the receiver operating characteristic curve; CV, cross-validation; LOPO-CV, leave-one-participant-out cross-validation; LOOCV, leave-one-out cross-validation; FEIT, facial emotion identification task.

6. **Supplementary Table S6.** Model performance across three feature settings in the **exploratory analyses.**

Feature setting	Model	Accuracy	ASD Recall	F1-Score	AUC
Static statistical features	KNN	0.71 ± 0.18	0.65 ± 0.37	0.63 ± 0.23	0.92 ± 0.06
	SVM	0.66 ± 0.15	0.75 ± 0.25	0.66 ± 0.11	0.84 ± 0.18
	LR	0.64 ± 0.14	0.70 ± 0.32	0.61 ± 0.14	0.81 ± 0.21
	XGBoost	0.71 ± 0.12	0.60 ± 0.33	0.61 ± 0.21	0.83 ± 0.17
	RF	0.73 ± 0.14	0.70 ± 0.32	0.66 ± 0.21	0.83 ± 0.14
Early feature concatenation	BiLSTM	0.75 ± 0.09	0.65 ± 0.33	0.66 ± 0.17	0.88 ± 0.09
	CNN	0.73 ± 0.13	0.50 ± 0.18	0.62 ± 0.19	0.89 ± 0.11
	TCN	0.75 ± 0.09	0.70 ± 0.27	0.69 ± 0.17	0.89 ± 0.08
	CNN–LSTM	0.78 ± 0.15	0.65 ± 0.28	0.70 ± 0.23	0.87 ± 0.10
Dynamic and static multiscale fusion	BiLSTM	0.76 ± 0.05	0.70 ± 0.27	0.71 ± 0.06	0.89 ± 0.09
	CNN	0.80 ± 0.09	0.75 ± 0.18	0.77 ± 0.09	0.86 ± 0.13
	TCN	0.78 ± 0.08	0.70 ± 0.21	0.73 ± 0.10	0.88 ± 0.10
	CNN–LSTM	0.80 ± 0.10	0.80 ± 0.21	0.78 ± 0.08	0.89 ± 0.10

Note. Values are reported as mean ± standard deviation across the five repeated participant-level evaluations. These results are from the exploratory analyses using the fixed seven-feature specification derived from the full dataset.

7. **Supplementary Table S7.** Participant-level classification performance, confusion matrices, and calibration results from the **exploratory analyses** of the CNN–LSTM multiscale fusion model.

Evaluation	TN	FP	FN	TP	Specificity	Precision	NPV	Brier score
**Repeated holdout Split 1**	**5**	**0**	**1**	**3**	**1.00**	**1.00**	**0.83**	**0.146**
**Repeated holdout Split 2**	**5**	**0**	**2**	**2**	**1.00**	**1.00**	**0.71**	**0.150**
**Repeated holdout Split 3**	**2**	**3**	**0**	**4**	**0.40**	**0.57**	**1.00**	**0.211**
**Repeated holdout Split 4**	**3**	**2**	**0**	**4**	**0.60**	**0.67**	**1.00**	**0.188**
**Repeated holdout Split 5**	**5**	**0**	**1**	**3**	**1.00**	**1.00**	**0.83**	**0.130**
**Repeated holdout Mean ± SD**	**-**	**-**	**-**	**-**	**0.80 ± 0.28**	**0.85 ± 0.21**	**0.88 ± 0.12**	**0.165 ± 0.034**
**Original exploratory LOPO sensitivity analysis**	**21**	**5**	**3**	**15**	**0.81**	**0.75**	**0.88**	**0.168**

Note. ASD was treated as the positive class and TD as the negative class. TN, true negative; FP, false positive; FN, false negative; TP, true positive; NPV, negative predictive value. Each test split in the repeated participant-level evaluation contained five TD and four ASD participants. Brier scores were calculated from participant-level predicted probabilities for the ASD class.

8. **Supplementary Table S8.** Approximate descriptive 95% confidence intervals for model performance metrics from the **exploratory analyses** across four feature settings and five repeated participant-level evaluations.

Feature setting	Model	Accuracy 95% CI	ASD Recall 95% CI	F1-score 95% CI	AUC 95% CI
Static statistical features	KNN	[0.45, 0.88]	[0.20, 0.93]	[0.33, 0.85]	[0.81, 0.97]
	SVM	[0.46, 0.82]	[0.36, 0.94]	[0.51, 0.78]	[0.50, 0.97]
	LR	[0.46, 0.79]	[0.26, 0.94]	[0.43, 0.76]	[0.44, 0.96]
	XGBoost	[0.54, 0.83]	[0.21, 0.89]	[0.34, 0.82]	[0.52, 0.96]
	RF	[0.53, 0.87]	[0.26, 0.94]	[0.38, 0.86]	[0.59, 0.94]
Dynamic sequences	BiLSTM	[0.49, 0.73]	[0.40, 0.89]	[0.48, 0.74]	[0.37, 0.72]
	CNN	[0.63, 0.85]	[0.40, 0.89]	[0.56, 0.83]	[0.66, 0.91]
	TCN	[0.53, 0.84]	[0.48, 0.91]	[0.50, 0.83]	[0.68, 0.91]
	CNN–LSTM	[0.62, 0.89]	[0.28, 0.90]	[0.40, 0.88]	[0.70, 0.92]
Early feature concatenation	BiLSTM	[0.62, 0.84]	[0.23, 0.92]	[0.43, 0.83]	[0.72, 0.95]
	CNN	[0.54, 0.86]	[0.29, 0.71]	[0.37, 0.82]	[0.67, 0.97]
	TCN	[0.62, 0.84]	[0.32, 0.92]	[0.45, 0.86]	[0.75, 0.96]
	CNN–LSTM	[0.54, 0.91]	[0.29, 0.90]	[0.37, 0.90]	[0.69, 0.95]
Dynamic and static multiscale fusion	BiLSTM	[0.69, 0.82]	[0.32, 0.92]	[0.63, 0.78]	[0.72, 0.96]
	CNN	[0.67, 0.89]	[0.48, 0.91]	[0.64, 0.86]	[0.62, 0.96]
	TCN	[0.67, 0.86]	[0.40, 0.89]	[0.59, 0.84]	[0.69, 0.96]
	CNN–LSTM	[0.65, 0.90]	[0.44, 0.95]	[0.67, 0.86]	[0.69, 0.97]

Note. Values represent approximate descriptive 95% confidence intervals for performance metrics across the five repeated participant-level evaluations.

9. **Supplementary Table S9.** Paired performance differences and 95% confidence intervals between multiscale fusion and early feature concatenation in **the exploratory analyses** across the five matched participant-level splits.

Model	Accuracy[95% CI]	ASD Recall[95% CI]	F1-Score[95% CI]	AUC[95% CI]
BiLSTM	+0.01<br>[−0.10, 0.10]	+0.05<br>[−0.41, 0.51]	+0.05<br>[−0.19, 0.26]	+0.01<br>[−0.02, 0.04]
CNN	+0.07<br>[−0.06, 0.19]	+0.25<br>[−0.10, 0.70]	+0.15<br>[−0.07, 0.40]	-0.03<br>[−0.10, 0.08]
TCN	+0.03<br>[−0.13, 0.17]	+0.00<br>[−0.38, 0.38]	+0.04<br>[−0.27, 0.34]	-0.01<br>[−0.15, 0.13]
CNN-LSTM	+0.02<br>[−0.09, 0.14]	+0.15<br>[−0.13, 0.43]	+0.08<br>[−0.11, 0.28]	+0.02<br>[−0.05, 0.09]

Note: Δ represents the mean performance difference between multiscale fusion and early feature concatenation (multiscale fusion − early feature concatenation). Values in brackets indicate 95% confidence intervals for the paired differences across the five matched participant-level splits.

10. **Supplementary Table S10.** Participant-level numbers of total and valid trials after gaze-data quality screening.

Group	Participant	Total	Valid
ASD	01	32	27
ASD	02	32	22
ASD	03	32	10
ASD	04	32	5
ASD	05	32	13
ASD	06	32	13
ASD	07	32	6
ASD	08	32	15
ASD	09	32	22
ASD	10	32	17
ASD	11	32	5
ASD	12	32	14
ASD	13	32	11
ASD	14	32	28
ASD	15	32	11
ASD	16	32	6
ASD	17	32	6
ASD	18	32	8
**ASD total**	—	**576**	239
TD	01	32	17

Group	Participant	Total	Valid
TD	02	32	12
TD	03	32	24
TD	04	32	15
TD	05	32	5
TD	06	32	16
TD	07	32	21
TD	08	32	7
TD	09	32	14
TD	10	32	17
TD	11	32	10
TD	12	32	14
TD	13	32	23
TD	14	32	20
TD	15	32	9
TD	16	32	12
TD	17	32	23
TD	18	32	18
TD	19	32	10
TD	20	32	16
TD	21	32	17
TD	22	32	10
TD	23	32	16
TD	24	32	17
TD	25	32	9
TD	26	32	9
**TD total**	—	**832**	381
**Overall**	—	**1408**	620

Note. Each participant completed 32 experimental trials. Trials with an effective gaze-sampling proportion below 65% were excluded from subsequent analysis. A total of 620 valid trials were retained after gaze-data quality screening. The excluded-trial proportions were 58.5% (337/576) for the ASD group and 54.2% (451/832) for the TD group.

11. **Supplementary Table S11.** Split-level participant classification performance of traditional machine-learning and CNN–LSTM models under training-only static feature selection.

Feature setting	Model	Split	Accuracy	ASD Recall	Specificity	Precision	NPV	F1-Score	AUC	Confusion Matrix
Static statistical features	KNN	1	0.778	1.000	0.600	0.667	1.000	0.800	1.000	(3, 2, 0, 4)
		2	0.667	0.500	0.800	0.667	0.667	0.571	0.850	(4, 1, 2, 2)
		3	0.778	0.500	1.000	1.000	0.714	0.667	0.550	(5, 0, 2, 2)
		4	0.889	1.000	0.800	0.800	1.000	0.889	0.900	(4, 1, 0, 4)
		5	0.556	0.250	0.800	0.500	0.571	0.333	0.750	(4, 1, 3, 1)
		Mean ± SD	0.733 ± 0.127	0.650 ±0.335	0.800 ± 0.141	0.727 ± 0.186	0.790 ±0.198	0.652 ± 0.216	0.810 ±0.171	—
Static statistical features	SVM	1	0.889	1.000	0.800	0.800	1.000	0.889	1.000	(4, 1, 0, 4)
		2	0.667	0.250	1.000	1.000	0.625	0.400	1.000	(5, 0, 3, 1)
		3	0.444	0.500	0.400	0.400	0.500	0.444	0.550	(2, 3, 2, 2)
		4	0.889	1.000	0.800	0.800	1.000	0.889	0.950	(4, 1, 0, 4)
		5	0.778	0.750	0.800	0.750	0.800	0.750	0.750	(4, 1, 1, 3)
		Mean ± SD	0.733 ± 0.186	0.700 ±0.326	0.760 ± 0.219	0.750 ± 0.218	0.785 ±0.223	0.674 ± 0.238	0.850 ±0.197	—
Static statistical features	LR	1	0.889	1.000	0.800	0.800	1.000	0.889	1.000	(4, 1, 0, 4)
		2	0.667	0.250	1.000	1.000	0.625	0.400	0.950	(5, 0, 3, 1)
		3	0.667	0.500	0.800	0.667	0.667	0.571	0.550	(4, 1, 2, 2)
		4	0.889	1.000	0.800	0.800	1.000	0.889	0.900	(4, 1, 0, 4)
		5	0.556	0.250	0.800	0.500	0.571	0.333	0.700	(4, 1, 3, 1)
		Mean ± SD	0.733 ± 0.149	0.600 ±0.379	0.840 ± 0.089	0.753 ± 0.185	0.773 ±0.210	0.617 ± 0.263	0.820 ±0.189	—
Static statistical features	RF	1	0.889	1.000	0.800	0.800	1.000	0.889	1.000	(4, 1, 0, 4)
		2	0.667	0.250	1.000	1.000	0.625	0.400	1.000	(5, 0, 3, 1)
		3	0.778	0.500	1.000	1.000	0.714	0.667	0.500	(5, 0, 2, 2)
		4	0.778	1.000	0.600	0.667	1.000	0.800	0.950	(3, 2, 0, 4)
		5	0.667	0.500	0.800	0.667	0.667	0.571	0.700	(4, 1, 2, 2)
		Mean ± SD	0.756 ± 0.093	0.650 ±0.335	0.840 ± 0.167	0.827 ± 0.167	0.801 ±0.184	0.665 ± 0.192	0.830 ±0.222	—
Static statistical features	XGBoost	1	0.889	1.000	0.800	0.800	1.000	0.889	1.000	(4, 1, 0, 4)
		2	0.667	0.250	1.000	1.000	0.625	0.400	0.950	(5, 0, 3, 1)
		3	0.556	0.500	0.600	0.500	0.600	0.500	0.500	(3, 2, 2, 2)
		4	0.667	0.500	0.800	0.667	0.667	0.571	0.800	(4, 1, 2, 2)
		5	0.667	0.500	0.800	0.667	0.667	0.571	0.650	(4, 1, 2, 2)
		Mean ± SD	0.689 ± 0.122	0.550 ±0.274	0.800 ± 0.141	0.727 ± 0.186	0.712 ±0.164	0.586 ± 0.183	0.780 ±0.208	—
Early feature	CNN–LSTM	1	0.889	0.750	1.000	1.000	0.833	0.857	0.950	(5, 0, 1, 3)

Feature setting	Model	Split	Accuracy	ASD Recall	Specificity	Precision	NPV	F1-Score	AUC	Confusion Matrix
concatenation										
		2	0.667	0.250	1.000	1.000	0.625	0.400	0.600	(5, 0, 3, 1)
		3	0.778	0.500	1.000	1.000	0.714	0.667	0.700	(5, 0, 2, 2)
		4	0.889	1.000	0.800	0.800	1.000	0.889	0.850	(4, 1, 0, 4)
		5	0.556	0.500	0.600	0.500	0.600	0.500	0.650	(3, 2, 2, 2)
		Mean ± SD	0.756 ± 0.145	0.600 ±0.285	0.880 ± 0.179	0.860 ± 0.219	0.755 ±0.165	0.663 ± 0.215	0.750 ±0.146	—
Multiscale fusion	CNN–LSTM	1	0.889	0.750	1.000	1.000	0.833	0.857	0.950	(5, 0, 1, 3)
		2	0.667	0.500	0.800	0.667	0.667	0.571	0.700	(4, 1, 2, 2)
		3	0.778	0.500	1.000	1.000	0.714	0.667	0.550	(5, 0, 2, 2)
		4	0.778	1.000	0.600	0.667	1.000	0.800	0.900	(3, 2, 0, 4)
		5	0.778	0.500	1.000	1.000	0.714	0.667	0.700	(5, 0, 2, 2)
		Mean ± SD	0.778 ± 0.079	0.650 ±0.224	0.880 ± 0.179	0.867 ± 0.183	0.786 ±0.135	0.712 ± 0.115	0.760 ±0.164	—

Note. Static feature selection was performed independently using only the training participants within each repeated participant-level split. Each test split included nine participants, comprising four children with ASD and five TD children. All metrics were calculated at the participant level. Specificity, precision, and negative predictive value (NPV) were calculated from the participant-level classification results. Confusion matrices are reported in the order TN, FP, FN, and TP. Mean ± SD values summarize the five repeated participant-level evaluations.

12. **Supplementary Table S12**. Participant-level confusion matrices and additional classification metrics for the CNN–LSTM multiscale fusion model in the primary analyses.

Evaluation	TN	FP	FN	TP	Specificity	Precision	NPV
**Repeated holdout Split 1**	**5**	**0**	**1**	**3**	**1.00**	**1.00**	**0.83**
**Repeated holdout Split 2**	**4**	**1**	**2**	**2**	**0.80**	**0.67**	**0.67**
**Repeated holdout Split 3**	**5**	**0**	**2**	**2**	**1.00**	**1.00**	**0.71**
**Repeated holdout Split 4**	**3**	**2**	**0**	**4**	**0.60**	**0.67**	**1.00**
**Repeated holdout Split 5**	**5**	**0**	**2**	**2**	**1.00**	**1.00**	**0.71**
**Repeated holdout Mean ± SD**	**–**	**–**	**–**	**–**	**0.88 ± 0.18**	**0.87 ± 0.18**	**0.79 ± 0.13**
**Fully nested LOPO evaluation**	**19**	**7**	**4**	**14**	**0.73**	**0.67**	**0.83**

Note. These results were obtained from the primary analyses in which static feature selection was performed using only the training participants within each participant-level evaluation. ASD was treated as the positive class and TD as the negative class. TN, true negative; FP, false positive; FN, false negative; TP, true positive; NPV, negative predictive value. Each test split in the repeated participant-level evaluation contained five TD and four ASD participants. Specificity, precision, and NPV were calculated from the participant-level confusion matrix for each evaluation. In the fully nested LOPO evaluation, each participant was held out once and excluded from static feature selection and model development. The LOPO results were calculated from the predictions for all 44 held-out participants and are reported separately from the mean ± SD results of the five repeated participant-level evaluations.
